# Mycobacterium abscessus Strain Morphotype Determines Phage Susceptibility, the Repertoire of Therapeutically Useful Phages, and Phage Resistance

**DOI:** 10.1128/mBio.03431-20

**Published:** 2021-03-30

**Authors:** Rebekah M. Dedrick, Bailey E. Smith, Rebecca A. Garlena, Daniel A. Russell, Haley G. Aull, Vaishnavi Mahalingam, Ashley M. Divens, Carlos A. Guerrero-Bustamante, Kira M. Zack, Lawrence Abad, Christian H. Gauthier, Deborah Jacobs-Sera, Graham F. Hatfull

**Affiliations:** a Department of Biological Sciences, University of Pittsburgh, Pittsburgh, Pennsylvania, USA; University of Massachusetts Amherst

**Keywords:** *Mycobacterium abscessus*, bacteriophage therapy, bacteriophages

## Abstract

Mycobacterium abscessus is an opportunistic pathogen whose treatment is confounded by widespread multidrug resistance. The therapeutic use of bacteriophages against Mycobacterium abscessus infections offers a potential alternative approach, although the spectrum of phage susceptibilities among M. abscessus isolates is not known. We determined the phage infection profiles of 82 M. abscessus recent clinical isolates and find that colony morphotype—rough or smooth—is a key indicator of phage susceptibility. None of the smooth strains are efficiently killed by any phages, whereas 80% of rough strains are infected and efficiently killed by at least one phage. The repertoire of phages available for potential therapy of rough morphotype infections includes those with relatively broad host ranges, host range mutants of Mycobacterium smegmatis phages, and lytically propagated viruses derived from integrated prophages. The rough colony morphotype results from indels in the glycopeptidolipid synthesis genes *mps1* and *mps2*, negating reversion to smooth as a common route to phage resistance. Resistance is thus rare, and although mutations in polyketide synthesis, *uvrD2*, and *rpoZ* can confer resistance, these likely also impair survival *in vivo*. The expanded therapeutic repertoire and the resistance profiles show that small cocktails or single phages could be suitable for controlling infections with rough strains.

## Abstract (precis)

Mycobacterium abscessus infections in cystic fibrosis patients are challenging to treat due to widespread antibiotic resistance. The therapeutic use of lytic bacteriophages presents a new potential strategy, but the great variation among clinical M. abscessus isolates demands determination of phage susceptibility prior to therapy.

## INTRODUCTION

Nontuberculous mycobacteria (NTM) are frequent pathogens of cystic fibrosis (CF) and bronchiectasis patients (1, 2). They are commonly refractory to treatment due to widespread antibiotic resistance and antibiotic toxicity over the required long treatment regimens (2–4). Among the NTMs, Mycobacterium abscessus is prevalent in CF patients, can greatly diminish lung function, and is a negative factor for lung transplantation (5, 6). There is an evident need for alternative treatments to control these infections.

There are three common subspecies of M. abscessus, subsp. *abscessus*, subsp. *bolletii*, and subsp. *massiliense* (7). M. abscessus subsp. *abscessus* is the most the common in CF patients, and genomic analysis shows that a high proportion of these strains form a clade of closely related strains that includes the ATCC 19977 type strain (8). Two distinct colony morphotypes of M. abscessus are observed, forming smooth (S) or rough (R) colonies on solid medium (9). The S strains generally are thought to be less virulent, due to the prevalence of surface glycopeptidolipids (GPLs) recognized by the host immune system (10–12). Rough strains are strongly depleted for GPLs, escape immune recognition more efficiently, and are more virulent (13). Smooth-to-rough transition occurs by interruption of GPL synthesis or localization—including mutations in *mmpL4*, which is required for GPL transport across the inner membrane—and mutation or silencing of *mps1*, *mps2*, and *gap* (9, 11, 14). Smooth-to-rough transitions are often nonreversible, although temperature-dependent variation has been reported for one variant (11, 15, 16).

The therapeutic use of bacteriophages may provide an alternative treatment strategy for NTM infections. A three-phage cocktail administered intravenously in a 15-year-old CF patient with a disseminated M. abscessus infection following a bilateral lung transplant showed substantial improvement and alleviation of infection (17). Whether phage interventions are useful in other patients with similar infections is unclear because of the extensive genetic variation among M. abscessus strains (18, 19).

## RESULTS

### Collection of Mycobacterium abscessus clinical isolates.

**FIG 1:**
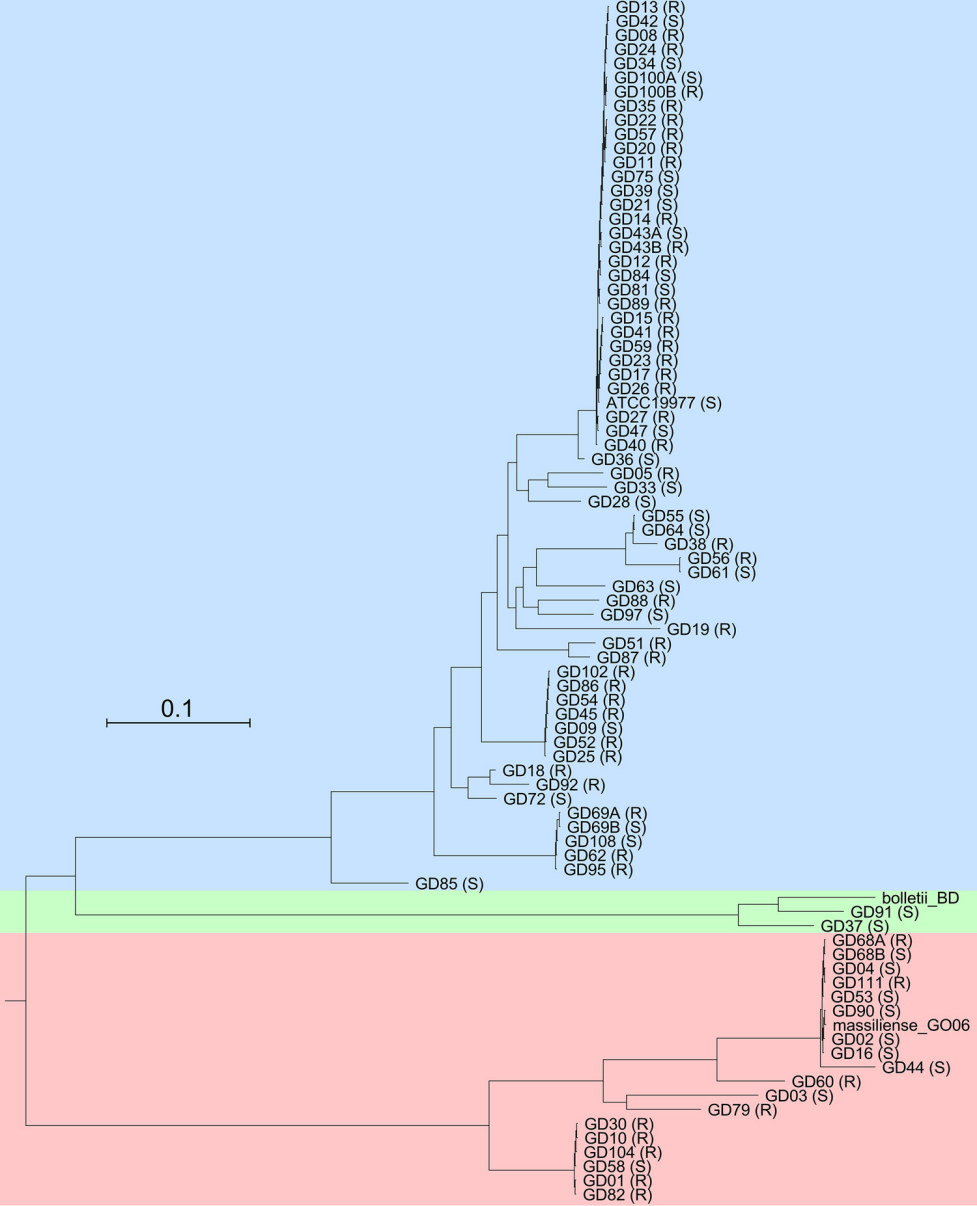
M. abscessus phylogeny. Phylogenetic relationships of M. abscessus clinical isolates based on 3,682,630 (78%) conserved nucleotide positions in all strains. Subspecies are shaded blue, green, and red for M. abscessus subsp. *abscessus*, M. abscessus subsp. *bolletii*, and M. abscessus subsp. *massiliense*, respectively. The rough (R) or smooth (S) morphotype is indicated after the strain name. Scale corresponds to 0.1 substitutions per position.

**FIG 2:**
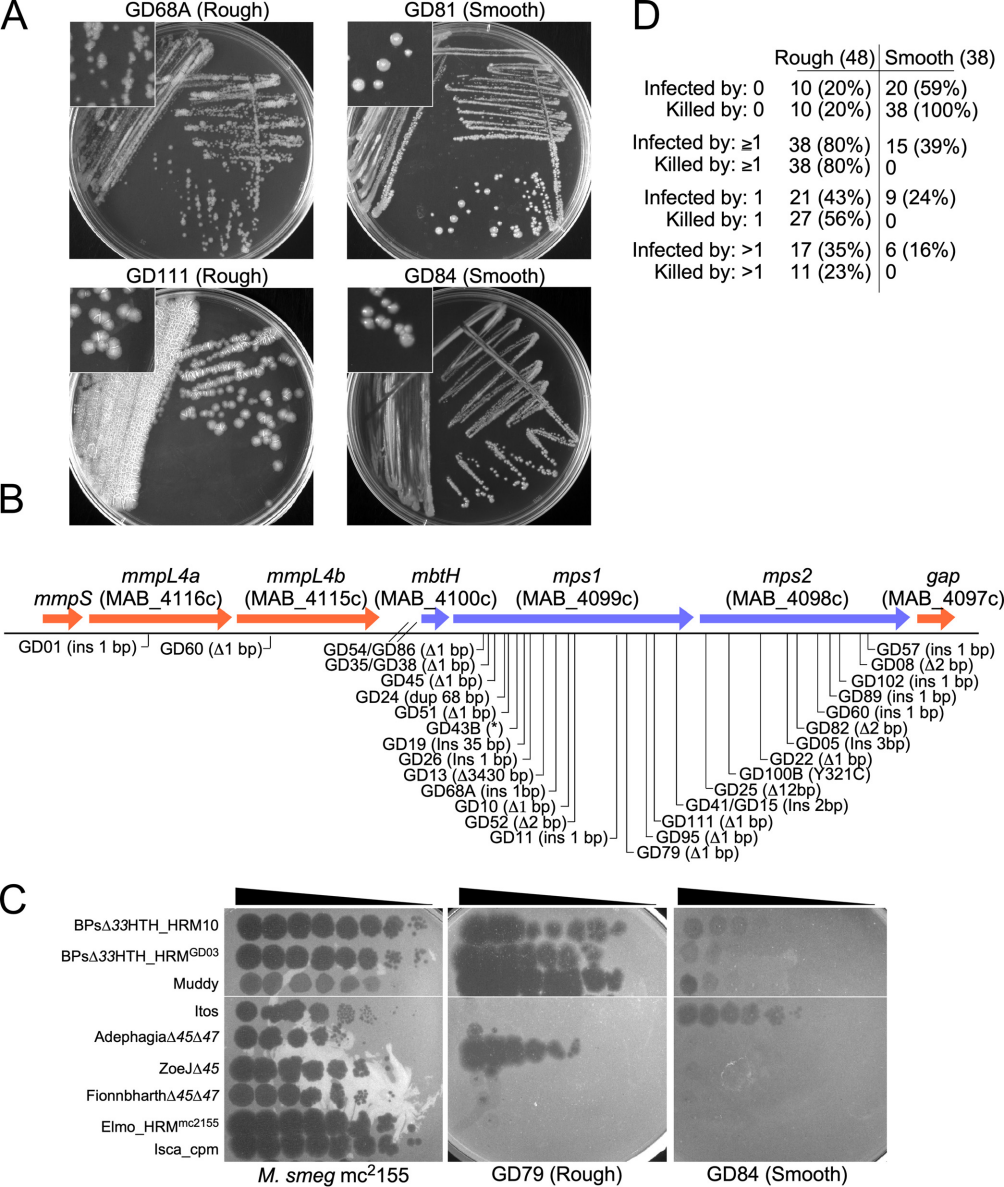
Smooth and rough M. abscessus morphotypes. (A) Examples of strain colony morphotypes showing strains GD68A and GD111 (both rough) and GD81 and GD84 (both smooth) growing on solid medium. Insets show magnified view of colonies. (B) Mutations contributing to rough colony morphotypes. Rough strain sequences were compared with ATCC 19977 genes involved in GPL synthesis (as shown) and those with small (1 to 2 bp) insertions or deletions are indicated. One mutation (GD100B) is a single base substitution in *mps2* and is the sole difference from its smoother counterpart, GD100A. A large spectrum of mutations is observed, although three pairs of rough strains (e.g., GD35 and GD38) have the same mutations. See Table S2 for details. (C) Plaque assay showing infection of M. smegmatis mc^2^155, M. abscessus GD79 (rough), and M. abscessus GD84 (smooth), as indicated. Ten-fold serial dilutions of phages were spotted from left to right on lawns of each strain, as indicated. (D) Phage susceptibilities of rough and smooth morphotype strains. The numbers of each strain morphotype that are not infected or killed by any phage tested (0), by at least one phage (≥1), by only a single phage (1), or by 2 or more phages (>1), as indicated, are shown. See Fig. 3 legend for details. The total number of strains (86) includes four strain pairs (e.g., A, B) each from a single patient source.

Following the successful treatment of a disseminated M. abscessus infection with a bacteriophage cocktail (17), we received 82 M. abscessus strains (designated GD01 to GD111; Table 1) from 78 different patients for characterization of phage susceptibilities; 54 (69%) of these are from within the United States and the others are from 10 different other countries (Table 1). We scored the colony morphotypes of each strain as being either rough (R) or smooth (S) (Table 1; Fig. 1, Fig. 2A); 48 (58.5%) have R morphotypes (Table 1). One-half of the strains are from patients with cystic fibrosis, and among these a similar proportion of strains (64%) are rough (Table 1). Smooth morphotype strains are less virulent than rough strains in some assays (20), but overall the smooth and rough profiles of these strains is similar to those reported previously (21, 22).

**TABLE 1:** Properties of Mycobacterium abscessus clinical isolates

Strain^*a*^	Origin^*b*^	CF^*c*^	Ssp^*d*^	R/S^*e*^	Seq^*f*^	Prophages^*g*^	Plasmids^*h*^
GD01	London, UK	Y	m	R	Com	None	None
GD02	London, UK	Y	m	R	WGS	prophiGD02-1 (MabN), prophiGD02-2 (MabA2)	pGD02 (pF)
GD03	Seattle, WA	N	m	S	WGS	prophiGD03-1 (MabG)	None
GD04	Los Angeles, CA	Y	m	S	WGS	prophiGD04-1 (MabE1)	None
GD05	Winston-Salem, NC	N	a	R	Com	prophiGD05-1 (MabD), prophiGD05-2 (MabH), prophiGD05-3 (MabM)	None
GD08	Indianapolis, IN	Y	a	R	WGS	prophiGD08-1 (MabA1), prophiGD08-2 (MabB), prophiGD08-3 (MabF)	pGD08 (pA)
GD09	Netherlands	Y	a	S	WGS	prophiGD09-1 (MabE1)	None
GD10	Pittsburgh, PA	N	m	R	WGS	prophiGD10-1 (MabA1)	pGD10 (pSin)
GD11	Pittsburgh, PA	Y	a	R	WGS	prophiGD11-1 (MabA1), prophiGD11-2 (MabB), phophiGD11-3 (MabF)	None
GD12	Pittsburgh, PA	NA	a	R	WGS	prophiGD12-1 (MabA1), prophiGD12-2 (MabD)	None
GD13	Pittsburgh, PA	NA	a	R	WGS	prophiGD13-1 (MabA1), prophiGD13-2 (MabC)	pGD13 (Sin)
GD14	Pittsburgh, PA	NA	a	R	WGS	prophiGD14-1 (MabA1), prophiGD14-2 (MabD)	None
GD15	Pittsburgh, PA	NA	a	R	WGS	prophiGD15-1 (MabA1)	None
GD16	St Louis, MO	Y	m	S	WGS	prophiGD16-1 (MabB), prophiGD16-2 (MabA2)	pGD16 (pH)
GD17	Durham, NC	Y	a	R	Com	prophiGD17-1 (MabD), prophiGD17-2 (MabA1)	None
GD18	San Diego, CA	N	a	S	WGS	None	pGD18 (pB)
GD19	San Diego, CA	Y	a	R	Com	None	pGD19 (pD)
GD20	Durham, NC	Y	a	R	Com	prophiGD20-1 (MabA1)	None
GD21	Baton Rouge, LA	N	a	S	Com	prophiGD21-1 (MabB), prophiGD21-2 (MabA1), prophiGD21-3 (MabG), prophiGD21-4 (MabJ)	pGD21-1 (pSin), pGD21-2 (pSin)
GD22	Pittsburgh, PA	Y	a	R	Com	prophiGD22-1 (MabA1)	pGD22-1 (pSin), pGD22-2 (pC)
GD23	St. Louis, MO	NA	a	R	WGS	prophiGD23-1 (MabA1)	pGD23 (pB)
GD24	Genoa, Italy	Y	a	R	WGS	prophiGD24-1 (MabA1), prophiGD24-2 (MabG), prophiGD24-3 (MabJ)	pGD24 (pC)
GD25	Anchorage, AL	Y	a	R	Com	prophiGD25-1 (MabE1)	pGD25-1 (pF), pGD25-2 (pG), pGD25-3 (pSin)
GD26	Pittsburgh, PA	Y	a	R	Com	prophiGD26-1 (MabA1)	None
GD27	Boston, MA	Y	a	R	WGS	prophiGD27-1 (MabA1)	None
GD28	Durham, NC	NA	a	S	WGS	None	None
GD30	Durham, NC	NA	m	R	WGS	prophiGD30-1 (MabA1)	None
GD33	Illes Balears, Spain	Y	a	S	WGS	prophiGD33-1 (MabC)	pGD33 (pE)
GD34	Boston, MA	N	a	S	WGS	prophiGD34-1 (MabA1), prophiGD34-2 (MabB)	pGD34 (pC)
GD35	New York, NY	Y	a	R	WGS	prophiGD35-1 (MabA1)	None
GD36	Vancouver, BC, Canada	N	a	S	WGS	prophiGD36-1 (MabA1), prophiGD36-2 (MabH)	pGD36-1 (pB), pGD36-2 (pE)
GD37	Vancouver, BC, Canada	N	b	S	WGS	None	None
GD38	Hamilton, ON, Canada	NA	a	R	Com	None	None
GD39	Hartford, CT	Y	a	S	WGS	prophiGD39-1 (MabA1), prophiGD39-2 (MabC)	pGD39 (pC)
GD40	NSW, Australia	Y	a	R	WGS	prophiGD40-1 (MabA1)	None
GD41	Pittsburgh, PA	Y	a	R	Com	prophiGD41-1 (MabA1)	None
GD42	Pittsburgh, PA	Y	a	S	Com	prophiGD42-1 (MabA1), prophiGD42-2 (MabB), prophiGD42-3 (MabC)	pGD42-1 (pB), pGD42-2 (pA)
GD43A	NSW, Australia	Y	a	S	Com	prophiGD43A-1 (MabA1), prophiGD43A-2 (MabB), prophiGD43A-3 (MabC), prophiGD43A-4 (MabJ), prophiGD43A-5 (MabK), prophiGD43A-6 (MabL)	None
GD43B	NSW, Australia	Y	a	R	Com	prophiGD43B-1 (MabL), prophiGD43B-2 (MabK), prophiGD43B-3 (MabA1), prophiGD43B-4 (MabJ)	None
GD44	Winnipeg, Canada	N	m	S	WGS	prophiGD44-1 (MabC)	None
GD45	Miami, FL	Y	a	R	WGS	prophiGD45-1 (MabE1)	pGD45-1 (pG), pGD45-2 (pD)
GD47	Dallas, TX	N	a	S	WGS	prophiGD47-1 (MabA1)	pGD47 (pB)
GD51	Portland, OR	Y	a	R	WGS	prophiGD51-1 (MabC), prophiGD51-2 (MabP)	pGD51 (pSin)
GD52	Sheffield, UK	Y	a	R	WGS	prophiGD52-1 (MabC), prophiGD52-2 (MabE1)	pGD52 (pSin)
GD53	Lebanon, NH	Y	m	S	WGS	prophiGD53-1 (MabE1), prophiGD53-2 (MabK), prophiGD53-3 (MabN)	None
GD54	NSW, Australia	Y	a	R	Com	prophiGD54-1 (MabE1), prophiGD54-2 MabI)	pGD54 (pF)
GD55	Cincinnati, OH	Y	a	S	WGS	prophiGD55-1 (MabL)	pGD55 (pSin)
GD56	San Jose, CA	NA	a	R	WGS	prophiGD56-1 (MabC), prophiGD56-2 (MabH)	None
GD57	Barcelona, Spain	Y	a	R	Com	prophiGD57-1 (MabC), prophiGD57-2 (MabA1)	None
GD58	Chapel Hill, NC	NA	m	S	WGS	prophiGD58-1 (MabG), prophiGD58-2 (MabA1)	pGD58 (pH)
GD59	Chapel Hill, NC	NA	a	R	Com	prophiGD59-1 (MabA1)	None
GD60	San Diego, CA	N	m	R	WGS	prophiGD60-1 (MabL)	None
GD61	San Jose, CA	NA	a	S	WGS	prophiGD61-1 (MabC), prophiGD61-2 (MabH)	None
GD62	Baton Rouge, LA	N	a	R	WGS	prophiGD62-1 (MabB), prophiGD62-2 (MabF), prophiGD62-3 (MabN)	pGD62-1 (pB), pGD62-2 (pC)
GD63	Memphis, TN	Y	a	S	WGS	None	None
GD64	Cambridge, UK	N	a	S	WGS	None	None
GD68A	Long Beach, CA	Y	m	R	Com	prophiGD68-1 (MabE1)	None
GD68B	Long Beach, CA	Y	m	S	Com	prophiGD68-1 (MabE1)	None
GD69A	Long Beach, CA	N	a	R	Com	prophiGD69-1 (MabN)	pGD69-1 (pB), pGD69-2 (pC)
GD69B	Long Beach, CA	N	a	S	Com	prophiGD69-1 (MabN)	pGD69-1 (pB), pGD69-2 (pC)
GD72	San Jose, CA	N	a	S	WGS	prophiGD72-1 (MabC)	pGD72 (pB)
GD75	Charleston, SC	N	a	S	WGS	prophiGD75-1 (MabJ), prophiGD75-2 (MabA1)	pGD75 (pC)
GD79	Bethesda, MD	N	m	R	WGS	prophiGD79-1 (MabQ)	None
GD81	Hartford, CT	Y	a	S	WGS	prophiGD81-1 (MabA1)	None
GD82	Baltimore, MD	N	m	R	WGS	prophiGD82-1 (MabC)	None
GD84	Dallas, TX	N	a	S	WGS	prophiGD84-1 (MabA1), prophiGD84-2 (MabD)	None
GD85	Ankara, Turkey	Y	a	S	WGS	None	pGD85 (pD)
GD86	Jerusalem, Israel	Y	a	R	WGS	prophiGD86-1 (MabI), prophiGD86-2 (MabE1)	pGD86-1 (pF), pGD86-2 (pG)
GD87	Jerusalem, Israel	Y	a	R	WGS	None	pGD87 (pB)
GD88	Jerusalem, Israel	Y	a	R	WGS	prophiGD88-1 (MabL)	None
GD89	Jerusalem, Israel	Y	a	R	WGS	prophiGD89-1 (MabB)	None
GD90	Jerusalem, Israel	Y	m	S	WGS	prophiGD90-1 (MabA2)	None
GD91	Bordeaux, France	N	b	S	Com	prophiGD91-1 (MabC), prophiGD91-2 (MabA3), prophiGD91-3 (MabO), prophiGD91-4 (MabE2)	None
GD92	Jacksonville, FL	N	a	R	WGS	None	None
GD95	Dallas, TX	N	a	R	WGS	prophiGD95-1 (MabG), prophiGD95-2 (MabN)	pGD95-1 (pB), pGD95-2 (pC)
GD97	Bizkaia, Spain	Y	a	S	WGS	None	None
GD100A	Philadelphia, PA	NA	a	S	Com	prophiGD100A-1 (MabA1), prophiGD100A-2 (MabC)	pGD100 (pC)
GD100B	Philadelphia, PA	NA	a	R	Com	prophiGD100A-1 (MabA1), prophiGD100A-2 (MabC)	pGD100 (pC)
GD102	Finland	Y	a	R	WGS	prophiGD102-1 (MabA1), prophiGD102 (MabE1)	pGD102-1 (pF), pGD102-2 (pG)
GD104	Knoxville, TN	N	m	R	WGS	prophiGD104-1 (MabA1), phiGD104-2 (MabC)	pGD104 (pSin)
GD108	Dallas, TX	NA	a	S	WGS	prophiGD108-1 (MabN)	pGD108 (pB)
GD111	Morton Grove, IL	N	m	R	WGS	prophiGD111-1 (MabE1)	None

a Strains are given a GDXX designation; multiple isolates from the same patient are designated GDXXA, GDXXB, etc.

b Strain origin indicates the location of the hospital or laboratory from which the strain was sent.

c Indication of whether strain was from a cystic fibrosis patient; Y, yes; N, no; NA, data not available.

d Subspecies derived from genome sequencing; a, *abscessus*; b, *bolletii*; m, *massiliense*.

e Colony morphotype is shown as R, rough or S, smooth.

f Genome sequencing with Illumina whole-genome sequencing (WGS) or Illumina and Nanopore to completion (Com).

g Prophages are designated prophiGDXX, with -1, -2-, etc. extensions denoting different prophages in the same strain.

h Plasmids are designated pGDXX, with -1, -2-, etc. extensions if there is more than one plasmid in the same strain.

10.1128/mBio.03431-20.2 TABLE S2 Genomic differences in smooth and rough Mycobacterium abscessus clinical isolates. Download Table S2, PDF file, 0.06 MB. Copyright © 2021 Dedrick et al. 2021 Dedrick et al. https://creativecommons.org/licenses/by/4.0/ This content is distributed under the terms of the Creative Commons Attribution 4.0 International license.

The M. abscessus genomes were sequenced either by Illumina technology to give whole-genome sequences (WGS) or to completion by the addition of Nanopore sequencing reads (Table 1; Table S1 in the supplemental material). Phylogenetic analysis of these strains together with the type-strains [M. abscessus subsp. *abscessus* ATCC 19977 (23), M. abscessus subsp. *bolletii* BD^T^ (24), and M. abscessus subsp. *massiliense* GO06 (25)], shows that 62 of the strains are subspecies *abscessus*, 18 are *massiliense*, and 2 are *bolletii* (Table 1, Fig. 1). The substantial genetic diversity is not surprising (8), and 50% of the strains form a closely related clade that includes the ATCC 19977 type strain (Fig. 1); the prevalence of this clade was noted previously (8). There are two smaller *massiliense* clades, one of which includes the previously characterized strain GO06 (25), and one that includes GD01, the strain from the previously phage-treated patient (17) (Fig. 1). Both of the subsp. *boletii* strains have S morphotypes, but R and S morphotypes are distributed throughout the rest of the phylogenetic spectrum (Table 1, Fig. 1, Fig. 2A). All of the strains are CRISPR-free.

10.1128/mBio.03431-20.1 TABLE S1 Sequencing details of Mycobacterium abscessus clinical isolates. Download Table S1, PDF file, 0.06 MB. Copyright © 2021 Dedrick et al. 2021 Dedrick et al. https://creativecommons.org/licenses/by/4.0/ This content is distributed under the terms of the Creative Commons Attribution 4.0 International license.

### Genotypes of smooth and rough colony morphotypes.

For four strains (GD43, GD68, GD69, and GD100), both S and R variants were recovered from the same sample and were sequenced to completion (Table 1). The R variants generally have several differences from the S variants, but these include mutations in GPL synthesis implicated in the rough morphotype (14). For example, rough strains GD43B and GD68A have single base differences in *mps1* (MAB_4099) relative to their smooth variants GD43A and GD68B, introducing nonsense and single base insertions, respectively (Fig. 2B; Table S2). Although the other R strains do not have a smooth counterpart, over 75% of them have mutations in *mps1* and *mps2* (MAB_4098), most commonly 1- to 2-bp insertions or deletions. One (GD60) also has a frameshift mutation in *mmpL4b* (MAB_4115) (Fig. 2B, Table S2). Only three instances of the same mutation in different strains were identified.

### Phage susceptibility profiles of M. abscessus clinical isolates.

**FIG 3:**
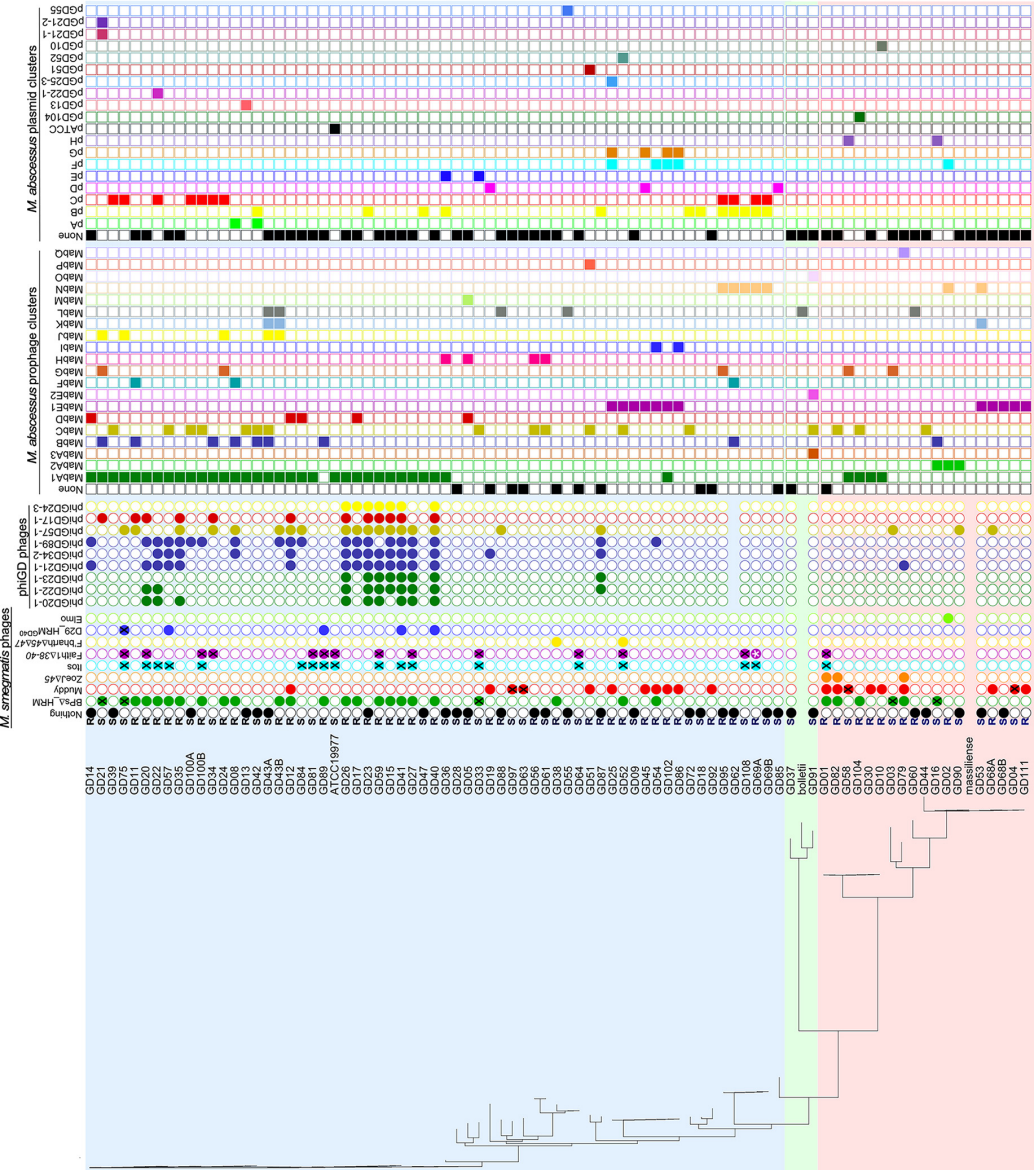
Properties and behaviors of M. abscessus clinical isolates. M. abscessus clinical isolates are shown with their phylogenetic relationships (as in Fig. 1), colony morphotype (R, rough; S, smooth) and susceptibilities to M. smegmatis phages (leftmost circles). Filled circles indicate either no phage infection (black) or efficiency of plating (EOP) relative to M. smegmatis of >0.1. Empty circles indicate no infection or an EOP relative to M. smegmatis of <0.1. Filled circles with an “X” reflect efficient infection (EOP >0.1) but poor killing; absence of circle, not tested. Filled circles with a white asterisk denote strains infected by HRM, but not the parental phage. The rightmost circles indicate susceptibilities to phiGD phages. Empty circles indicate no infection and filled circles reflect efficiency of plating relative to the original recipient strain of >0.01. To the right are squares indicating the presence of prophages and plasmids, with the cluster indicated at the top. Filled squares, presence of prophage or plasmid; empty squares, absence of prophage or plasmid.

We previously reported that M. abscessus strain GD01 is efficiently infected and killed by the phages Muddy, BPs, and ZoeJ, although these were the only phages identified from a screen of about 100 individual phages isolated on M. smegmatis (17). Few phages have been isolated on M. abscessus strains directly (17), and initial evaluation of several dozen M. smegmatis phages confirmed that many do not infect any of the clinical isolates; we therefore focused on eight of the most promising candidates (Fig. 1, Fig. 2C, Fig. 3): Muddy, BPs, ZoeJ, Itos, Faith1, Fionnbharth, D29, and Elmo (members of clusters/subclusters AB, G1, K1, L2, L2, K4, A2, and A3, respectively) (26, 27) or their derivatives, as described below.

The substantial variation in the infection profiles is striking (Fig. 3). Many different susceptibility combinations are observed and there is no evident correlation with the whole-genome phylogenies. Approximately 34% of the strains are not susceptible to any of the phages tested, spanning subspecies and clades (Fig. 3). There are several instances in which closely related strains of different origins have similar profiles, such as GD01 and GD82 from patients in the UK and US, respectively, which differ by only a few dozen nucleotide polymorphisms and have similar susceptibility profiles (Table 1, Fig. 3).

**FIG 4:**
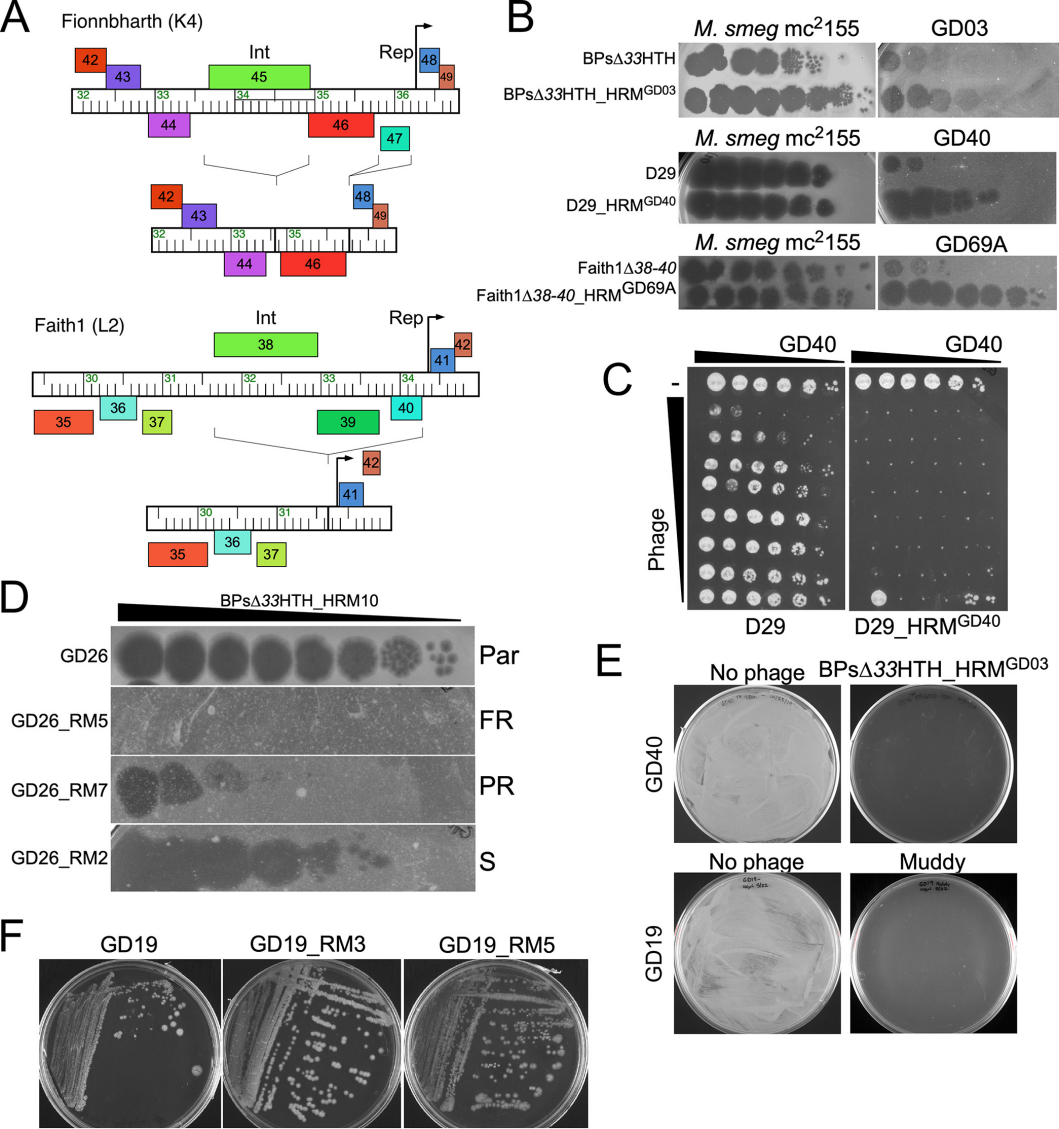
M. abscessus phage mutants and resistance. (A) Engineering lytic derivatives of phage Fionnbharth and Faith1. Central genomic regions are shown for each phage with rightward- and leftward-transcribed genes shown above and below the genome ruler, respectively. The subcluster K4 phage Fionnbharth genome was edited to remove the integrase (*int*) and repressor (*rep*) genes as indicated. The subcluster L2 phage Faith1 was edited to remove genes *38* to *40* including *int* and *rep*. (B) Ten-fold serial dilutions of host range mutants (HRM) of BPsΔ*33*HTH, D29, and Faith1Δ*38-40* were plated from left to right on M. smegmatis and the M. abscessus strain on which the HRM was isolated. (C) Killing assay of M. abscessus strain GD40 with phages D29 (left) and D29_HRM^GD40^ (right). Each column has 10-fold serial dilutions of M. abscessus GD40 (leftmost column, 3 × 10^5^ CFU), and each row has 10-fold serial dilutions of either phage D29 or D29_HRM^GD40^ (topmost row, 10^7^ PFU). Cells and phage were mixed and incubated for 48 h prior to plating on solid medium. (D) Phage susceptibility assay illustrating that strains recovered from BPsΔ*33*HTH_HRM10 challenge of GD26: Par, parental; FR, fully resistant GD26_RM5; PR, partially resistant GD26_RM7; and S, fully sensitive GD26_RM2. Spots are 10-fold serial dilutions of BPsΔ*33*HTH_HRM10 from left to right. (E) Efficient killing of M. abscessus strains GD40 and GD19 (as indicated) with phages BPsΔ*33*HTH_HRM^GD03^ or Muddy. Aliquots of 10^7^ CFU were incubated with or without phage (multiplicity of infection [MOI] 10) for 48 h and plated on solid medium. (F) Colony morphotypes of GD19 and Muddy-resistant mutants GD19_RM3 and GD19_RM5. GD19 and GD19_RM3 are rough, and GD19_RM5 is smooth.

Phages forming plaques on clinical isolates may not necessarily kill them efficiently, and we therefore determined this using challenge assays to measure bacterial survival (Fig. 4C and E, Table S3). There is a striking correlation between phage killing efficiency and strain colony morphotype. Of the 48 R strains, 38 (80%) are efficiently killed by at least one phage, 17 of which (35%) are killed by more than one (Fig. 2D). In contrast, none of the 38 S strains are killed, although a subset (21%) is infected by phages Faith1 and/or Itos, but neither kills the strains efficiently (Fig. 1, Fig. 2D). Surface GPLs on the smooth strains may be responsible for the poor phage infection, but it is unclear why lytic derivatives of Faith1 and Itos (see below) do not efficiently kill the S strains they infect (Fig. 2D, Fig. 3). This poor-killing phenotype is not constrained to the subcluster L2 phages and is also observed in some R and S strains with phages BPs, Muddy, and D29 (Fig. 2D, Fig. 3).

10.1128/mBio.03431-20.3 TABLE S3 M. abscessus survival following phage challenge. Download Table S3, PDF file, 0.1 MB. Copyright © 2021 Dedrick et al. 2021 Dedrick et al. https://creativecommons.org/licenses/by/4.0/ This content is distributed under the terms of the Creative Commons Attribution 4.0 International license.

### Expanding the phage repertoire by genome engineering.

Several of the potentially useful phages are temperate and form stable lysogens in M. smegmatis. Muddy is not evidently temperate, and efficiently kills most of the M. abscessus strains it infects. Itos, D29, and Elmo are not temperate, but are naturally lytic derivatives of temperate phages that lack the repressor gene, which is responsible for establishing and maintaining lysogeny. We previously described the engineering of both BPs and ZoeJ to construct lytic derivatives (17, 28), and we used similar approaches to construct lytic versions of Fionnbharth (i.e., FionnbharthΔ*45*Δ*47*) and Faith1 (i.e., Faith1Δ*38-40*) (Fig. 4A).

### Expanding the phage repertoire using host range mutants.

In screening strains, several different phenotypes of M. abscessus phage infection were observed. These include an absence of infection, an efficiency of plaquing (e.o.p.) of one relative to M. smegmatis, and killing of cells only at high phage concentrations (Fig. 2C). Sometimes, individual plaques are observed at high phage titers, arising from phenotypic escape of defense systems, such as restriction or genetic alteration that promotes efficient infection. We previously described a host range mutant (HRM) of phage BPsΔ*33*HTH (BPsΔ*33*HTH_HRM10) (17), and we successfully isolated another HRM of BPsΔ*33*HTH (BPsΔ*33*HTH_HRM^GD03^) using M. abscessus GD03 as the host (Fig. 4B). We similarly isolated HRMs of phages D29 and Faith1Δ*38-40* (Fig. 4B).

The lytic subcluster A2 phage D29 (29, 30) does not efficiently infect any M. abscessus strain, but an HRM was isolated on M. abscessus GD40, purified, and shown to efficiently infect GD57, GD89, and GD41, in addition to GD40 (Fig. 3, Fig. 4B). D29_HRM^GD40^ has three mutations relative to its immediate precursor, C12442A, T31726C, and A43657T, but reconstruction of the individual mutants demonstrated that the C12442A mutation conferring a T116N substitution in the capsid protein is responsible for the host range phenotype (Fig. 4B). Capsid mutations associated with phage host range are unusual but have been described to influence adsorption of ϕX174 (31). The potential therapeutic utility of D29_HRM^GD40^ is illustrated by its enhanced killing of strain GD40 relative to the D29 parent phage (Fig. 4C).

Faith1 does not efficiently infect M. abscessus GD69A but an HRM was isolated (Faith1Δ*38-40*_HRM^GD69A^) that does (Fig. 4B), although the HRM does not change its infection of other strains. Faith1Δ*38-40*_HRM^GD69A^ has a single mutation at G34936T conferring an early termination codon in gene *43* (E11*), whose function is unknown, but it is highly expressed in early lytic growth of its relative Crossroads (32). Faith1 gp43 is unlikely to be involved in cell surface interactions and may be triggering a host defense system to which the Faith1Δ*38-40*_HRM^GD69A^ mutant is able to escape.

### M. abscessus phage resistance.

Phage-susceptible strains were challenged with phage(s) in liquid cultures and 10^7^ cells plated on solid medium to recover survivors. Robust growth was observed for all of the smooth strains (>50% survival) reflecting very poor killing (Table S3). In contrast, 70% of the 75 combinations of phage and rough strains, using a total of 28 strains, yielded either no or very few survivors, which upon retesting were either fully or partially phage sensitive (Fig. 4D and E; Table S3). Only 12 of the 28 strains (43%) yielded any resistant mutants (Table S3). With the exception of mutant GD19_RM5, which converted to S, all of the resistant mutants retained their R morphotype (Fig. 4F). This is notable because reversion or suppression to smooth could be a simple route to resistance, but evidently is relatively uncommon due to the type of mutations conferring rough morphotypes (Fig. 2B). We note that no resistant strains for GD43B were recovered (Table S3), suggesting that reversion or suppression of the *mps1* nonsense mutation occurs below the detection limit of the assay.

Nine phage-resistant strains were sequenced and compared to their phage-sensitive parents (Table S4). GD19_RM5 (Fig. 4F) contains a wild-type allele of *mps1* (MAB_4099), and direct reversion of the 35-bp insertion in *mps1* (MAB_4099) has given both the smooth morphotype and resistance to Muddy (Fig. 2B, Fig. 4F). Two mutants (GD17_RM1 and GD22_RM4) have mutations in a type I polyketide synthase (MAB_0939), implicated in synthesis of trehalose polyphleates (33); related proteins are nonessential for M. tuberculosis growth *in vitro* but are important for virulence (34). Mutants GD22_RM1 and GD22_RM2 have mutations in the C-terminal HRDC domain of UvrD2 (MAB_3511), which is nonessential for M. tuberculosis growth (35). For GD26_RM4 and GD25_RM2, it is unclear which genes are implicated in resistance, either a 28.5-kbp deletion or multiple changes from its parent; however, both include genes implicated in virulence (Table S4). GD22_RM3 has lost plasmid pGD22-1 (36), which could impact phage infection.

10.1128/mBio.03431-20.4 TABLE S4 Genomic differences in phage-resistant mutants (RMs) of Mycobacterium abscessus clinical isolates. Download Table S4, PDF file, 0.04 MB. Copyright © 2021 Dedrick et al. 2021 Dedrick et al. https://creativecommons.org/licenses/by/4.0/ This content is distributed under the terms of the Creative Commons Attribution 4.0 International license.

Mutant GD19_RM3 (Fig. 4F) has a frameshift mutation (4-bp deletion) in *rpoZ* coding for the RNA polymerase omega subunit, and is the sole sequence difference from GD19; *rpoZ* is clearly not essential for M. abscessus growth, as reported for M. smegmatis (37). Deletion of *rpoZ* in M. smegmatis does not alter the GPL profile but it reduces both sliding motility and biofilm formation, and has a notable reduction in short-chain mycolates on the cell surface (37). Similar surface changes in GD19_RM3 may be responsible for the inability of phage Muddy to infect. None of the other strains described here have *rpoZ* mutations, and this RM characterization illustrates the multitude of mechanisms that alter phage susceptibility.

### Isolation and propagation of spontaneously released lytic phages.

**FIG 5:**
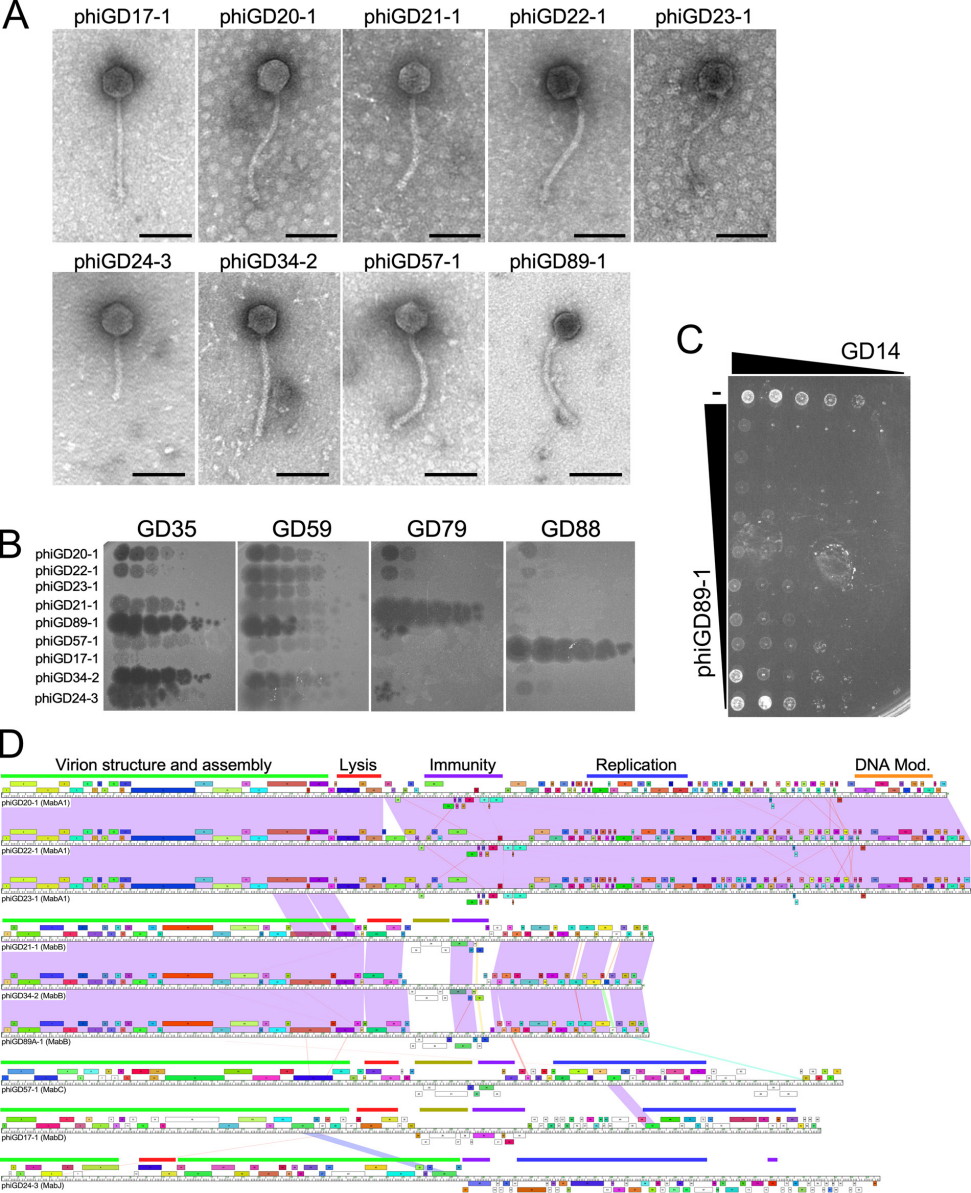
Lytically propagating phiGDxx phages. (A) Electron micrographs of nine lytically growing phages released by spontaneous prophage induction. Scale bar is 100 nm. (B) The nine lytically growing phages recovered from M. abscessus GD strains (as shown) were 10-fold serially diluted and spotted onto lawns of GD35, GD59, GD79, and GD88 to illustrate infection profiles. Full infection profiles are shown in Fig. 3. (C) Killing assay showing reduction in viability of M. abscessus GD14 by phiGD89-1. Configuration is as shown in Fig. 4C. (D) Genome maps of the nine lytically growing phages recovered from M. abscessus strains, with pairwise nucleotide sequence similarity shown as spectrum colored shading, with violet indicating closest similarity and red the least above a threshold BLASTN E value of 10^−4^. Genes are shown as boxes above (transcribed rightward) and below (transcribed leftward) each genome; boxes are colored according to the gene phamilies they are assigned (50). Slim bars above genome sequence identify categories of gene functions: green bar, structural genes; red bar, lysis cassette; purple bar, immunity cassette; blue bar, replication genes, orange bar, DNA modification genes; and olive bars, polymorphic toxin genes. Detailed genome maps for all phages are in Fig. S1 to S5 and Fig. S6 to S9 in the supplemental material (https://phagesdb.org/documents/categories/15/).

To expand the suite of phages with therapeutic potential, we searched for phages that are spontaneously released from the M. abscessus strains that form plaques on other strains. A screen of over 1,200 pairwise tests identified nine distinct phages (designated phiGDxx), corresponding to nine different donor and five recipient strains (Table 2). Each phage was purified on its recipient strain, sequenced, and annotated (Fig. 5, Table 2, Fig. S1 to S5, Fig. S6 to S9) (https://phagesdb.org/documents/categories/15/). All have features typical of temperate phages and their organizations and virion genes are consistent with their siphoviral morphologies (Fig. 5A). None grow on M. smegmatis, and none are closely related to previously described M. smegmatis phages (26, 27). Electron microscopy showed all nine phages have siphoviral morphologies (Fig. 5A). The phiGDxx phages are expected to be temperate and form turbid plaques, as they are derived from resident prophages. This is observed for most of the phages, but not for phiGD89-1, which forms clear plaques (Fig. 5B). phiGD89-1 differs from its close relatives phiGD21-1 and phiGD34-2 by several nucleotide differences immediately upstream of the early lytic genes, which may confer the clear-plaque phenotype. phiGD34-2 forms turbid plaques on most strains, but not on all, and the plaques appear clear on strains such as GD35 (Fig. 5B).

**TABLE 2:** Lytically propagated phages derived from Mycobacterium abscessus

Name^*a*^	Recipient^*b*^	Cluster^*c*^	Length (bp)^*d*^	ORFs^*e*^	Equivalent prophage^*f*^	Accession no.
phiGD20-1	GD35	MabA1	59,055	118	prophiGD20-1 (MabA1)	MW314858
phiGD22-1	GD40	MabA1	60,512	120	prophiGD22-1 (MabA1)	MW314856
phiGD23-1	GD40	MabA1	60,535	120	prophiGD23-1 (MabA1)	MW314855
phiGD21-1	GD41	MabB	40,735	63	prophiGD21-1 (MabB)	MW314857
phiGD34-2	GD41	MabB	40,005	63	prophiGD34-2 (MabB)	MW314853
phiGD89-1	GD89B	MabB	40,450	63	prophiGD89A-1 (MabB)	MW314851
phiGD57-1	GD35	MabC	52,563	72	prophiGD57-1 (MabC)	MW314852
phiGD17-1	GD40	MabD	51,185	88	prophiGD17-1 (MabD)	MW314859
phiGD24-3	GD17	MabJ	54,877	94	prophiGD24-3 (MabJ)	MW314854

a Phages are named according to the donor strains from which they were isolated.

b Recipient is the sensitive strain on which the phage was isolated on.

c Cluster designation (MabA, MabB, etc.), corresponds to the prophage cluster assignment.

d Phage genome length in base pairs (bp).

e Predicted numbers of open reading frames (ORFs).

f Name of prophage present in the donor strain corresponding to the lytically propagated phage. Cluster designation is shown in parentheses.

Each of the nine phages was tested for infection of the GDxx strains (Fig. 3). Phage phiGD89-1 infects about one-third of the R strains, most of which are in the large M. abscessus subsp. *abscessus* clade (Fig. 3, Fig. 5B). The closely related phiGD21-1 has a similar profile, although some strains, such as GD08, distinguish between the two phages (Fig. 3). Seven strains—four S type (GD34, GD84 GD90, and GD100A) and three R type (GD14, GD23, and GD88)—that are not killed efficiently by other phages are efficiently infected by at least one phiGD phage (Fig. 3), increasing the proportion of efficiently infected R strains to 85%. They also add a potential second phage to 10 other R strains that are only killed by a single phage. This expansion of the phage repertoire is illustrated by the ability of phiGD89-1 to kill GD14, for which no other phages have been identified (Fig. 3, Fig. 5C). The temperate phiGDxx phages will need to be engineered for obligatory lytic growth prior to therapeutic consideration. The phiGDxx genomes vary in length from 40 to 60 kbp and represent five distinct groups based on overall sequence relationships (Fig. 5D, Table 2); detailed genome maps are shown in Fig. S1 to S5 and Fig. S6 to S9 (https://phagesdb.org/documents/categories/15/).

10.1128/mBio.03431-20.5 FIG S1 Genome organization of phiGD20-1 (MabA1). The genome of phiGD20-1 is shown with predicted genes shown as boxes either above or below the genome indicating rightward- and leftward-transcription, respectively. Gene numbers are shown within each gene box. Phamily designations are shown above or below each gene, with the numbers of phamily members in parentheses; genes are colored according to the phamily designations. White boxes represent “orphams,” i.e., genes with no close relatives in this dataset. Phamily assignments were determined using Phamerator and database Actino_prophage (version 5). Predicted gene functions are indicated. Download FIG S1, PDF file, 0.4 MB. Copyright © 2021 Dedrick et al. 2021 Dedrick et al. https://creativecommons.org/licenses/by/4.0/ This content is distributed under the terms of the Creative Commons Attribution 4.0 International license.

10.1128/mBio.03431-20.6 FIG S2 Genome organization of phiGD21-1 (MabB). See Fig. S1 for details. Download FIG S2, PDF file, 0.2 MB. Copyright © 2021 Dedrick et al. 2021 Dedrick et al. https://creativecommons.org/licenses/by/4.0/ This content is distributed under the terms of the Creative Commons Attribution 4.0 International license.

10.1128/mBio.03431-20.7 FIG S3 Genome organization of phiGD57-1 (MabC). See Fig. S1 for details. Download FIG S3, PDF file, 0.2 MB. Copyright © 2021 Dedrick et al. 2021 Dedrick et al. https://creativecommons.org/licenses/by/4.0/ This content is distributed under the terms of the Creative Commons Attribution 4.0 International license.

10.1128/mBio.03431-20.8 FIG S4 Genome organization of phiGD17-1 (MabD). See Fig. S1 for details. Download FIG S4, PDF file, 0.3 MB. Copyright © 2021 Dedrick et al. 2021 Dedrick et al. https://creativecommons.org/licenses/by/4.0/ This content is distributed under the terms of the Creative Commons Attribution 4.0 International license.

10.1128/mBio.03431-20.9 FIG S5 Genome organization of phiGD24-3 (MabJ). See Fig. S1 for details. Download FIG S5, PDF file, 0.3 MB. Copyright © 2021 Dedrick et al. 2021 Dedrick et al. https://creativecommons.org/licenses/by/4.0/ This content is distributed under the terms of the Creative Commons Attribution 4.0 International license.

### M. abscessus prophages.

**FIG 6:**
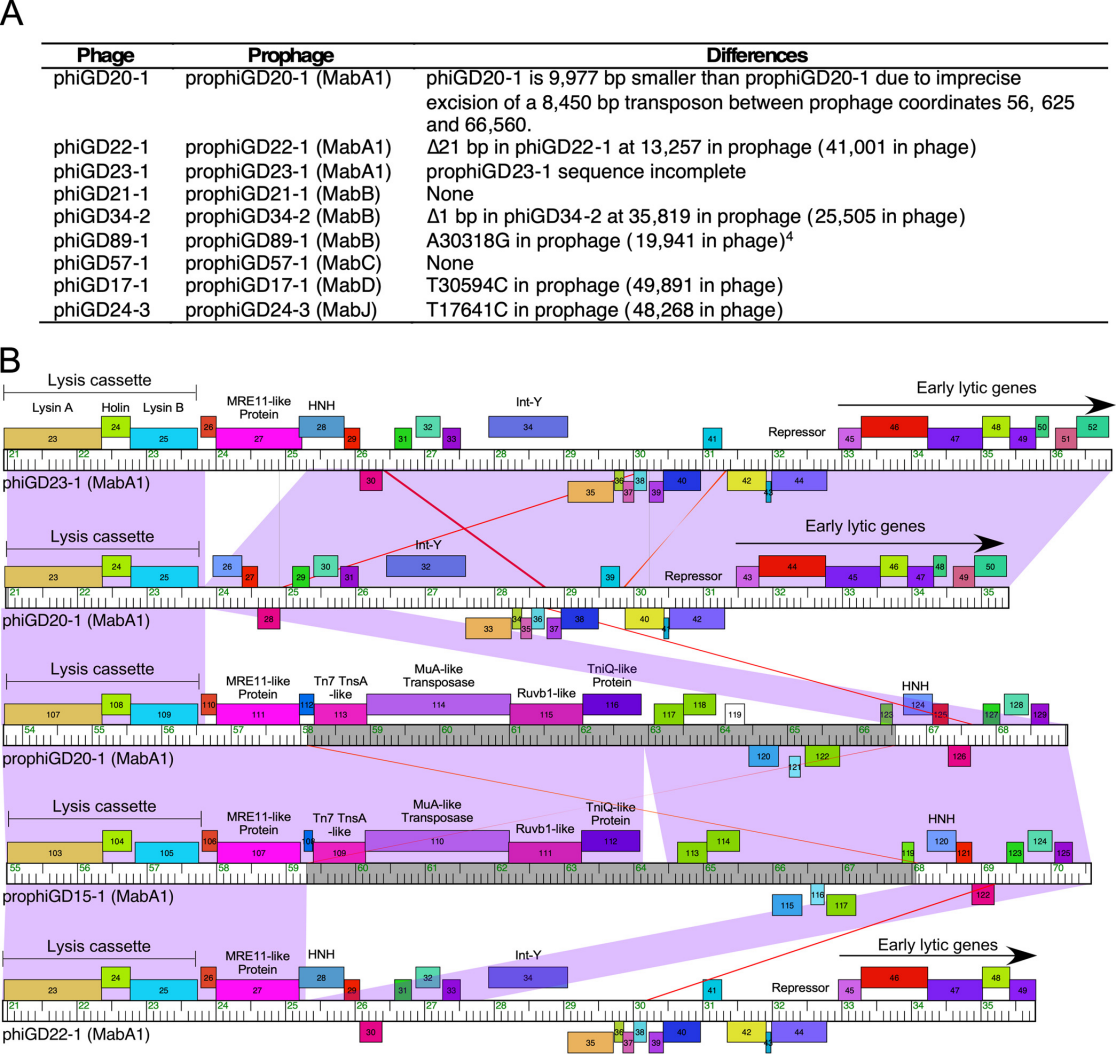
Comparison of induced phages and their cognate prophages. (A) Comparisons of phage (i.e., phiGDxx) and their cognate prophages (i.e., prophiGDxx) showing genomic differences; phages and prophages are named after the strain they were isolated from. The prophiGD23-1 sequence is incomplete in the WGS assembly and thus cannot be fully compared with phiGD23-1. In phiGD89-1, there are several differences in a region that is well conserved in other MabB phages, including phiGD21-1. Specifically, the sequence 5′-TGGACTACGGCTGAGCAGCA-TGCT (coordinates 30,519 to 30,542) replaces 5′-TGGGCTACGGCTGAGCAGTAGAACT. This is in the location of a predicted rightward early promoter and operator site, and likely inactivates lysogeny. (B) Comparison of phage and prophage genome segments, illustrating the transposon insertions (gray areas on genome rulers) in prophages prophiGD20-1 and prophiGD15-1. Genome annotations and comparisons are illustrated as in Fig. 5D. The alignment illustrates the transposon insertion (comparing phiGD22-1 and prophiGD15-1), and excision of the transposon and flanking gene in phiGD20-1. The transposons in prophiGD20-1 and prophiGD15-1 are closely related but inserted at targets 25 bp apart.

Using the phiGDxx sequences, we identified the cognate prophages (designated prophiGDxx) in the donor strains and extracted their complete prophage sequences (Table 1, Fig. 6). Genome comparisons showed that phages phiGD21-1 and phiGD57-1 are identical to their cognate prophages, and spontaneous induction involves simple excisive site-specific recombination between the *attL* and *attR* junctions. However, six of the phages differ from their prophages by one or more base substitutions and/or insert/deletions, which presumably occurred during induction or subsequent lytic growth (Fig. 6A). Curiously, prophiGD20-1 is 9,977 bp larger than phiGD20-1 and contains an 8,452-bp transposon insertion in an HNH-like gene, which is defined by 25-bp imperfect inverted repeats (IRs) and flanked by a 5-bp target duplication (Fig. 6B). Loss of the transposon—presumably a requirement for lytic growth due to DNA packaging constraints—has occurred by transposase-mediated imprecise excision between the rightmost IR junction and a location within prophiGD21-1 gene *110*; excision also removes an *Mre-11*-like gene which is evidently not required for lytic growth (Fig. 6B). A second closely related prophage (prophiGD15-1; see below) has a similar transposon inserted within the same HNH gene, 25 bp to the left of that in prophiGD20-1. Moreover, two additional prophages, prophiGD41-1 and prophiGD59-1, are identical to prophiGD15-1 with the same transposon insertions. The transposon is also present in M. abscessus subsp. *bolletii* CCUG, M. abscessus FLAC013, and M. abscessus UC22 (GenBank accession numbers AP014547, CP014955, and CP012044).

Bioinformatic analyses shows that prophages are abundant in the GD strains, as has been reported for other M. abscessus strains (38). These prophages, along with plasmids from these strains, are described in detail elsewhere (36) (Tables S5 and S6) (https://phagesdb.org/documents/categories/15/), and their inclusion in each strain is indicated in Fig. 3. Only 12 strains are prophage-free, and the 122 prophages and nine lytically growing phages (Table 2) are grouped into clusters (MabA to MabQ) according to their sequence relationships (Fig. 6A, Table S5). Because the phage susceptibility profiles do not correlate closely with nucleotide sequence-based phylogeny (Fig. 3), the prophage and plasmid contents are strong candidates for contributing to the phage infection profiles. The abundance and diversity of the M. abscessus mobilome confounds any simple elucidation of their roles. We note, however, that many of the prophages and plasmids code for type II toxin-antitoxin systems which can confer viral defense (36).

## DISCUSSION

The phage infection profiles of these M. abscessus strains illuminate both the opportunities and challenges in their therapeutic prospects. Smooth morphotype strains are problematic and not efficiently killed by any of the phages, and a surprisingly high proportion (36%) of strains are smooth, suggesting that they are important pathogens, but perhaps with pathologies distinct from rough strains. The observation that smooth strains with abundant surface GPLs are not efficiently killed by phages is in contrast to the finding that GPLs are required for M. smegmatis infection by phage I3 (39). Through extensive phage screening, genome engineering, host range mutant identifications, and induction of prophages, a relatively small set of phages were identified that can infect and kill 80% of the rough strains. The induced prophages require further engineering to remove genes for lysogeny and potential virulence genes, but are a promising source of new phages, increasing the number of rough strains targeted to about 85%. Until additional phages are identified that expand the repertoire to kill all of the rough strains, screening of individual strains is still required before therapeutic intervention because of the variable and unpredictable phage susceptibility profiles. The variability in phage infection is likely determined in large part by the impressive array of diverse prophages and plasmids in these M. abscessus clinical isolates.

With only a rather limited set of phages available for therapy—and a substantial proportion of rough strains are killed by only a single phage—the frequencies and mechanisms of phage resistance are key concerns. For most M. abscessus phage infections, no stably resistant survivors were isolated, and when resistant mutants were recovered, the mutations suggest there may be a fitness cost for *in vivo* growth. Furthermore, although reversion to a smooth morphotype is expected to contribute to resistance, this occurs only rarely, reflecting the predominance of mutations leading to rough morphotypes that occur only infrequently. In general, use of a single phage for therapy when antibiotic treatments are no longer available would seem a reasonable strategy. We recognize that some infections contain both rough and smooth M. abscessus variants and can vary by anatomical location and duration (14, 15, 22), raising the question as to whether phage-mediated killing of the resistant strain alone will provide any therapeutic effect. This is likely to vary considerably among patients, depending on the virulence capacity of the smooth variant.

Challenges in the therapeutic use of phages arise for many other bacterial pathogens other than M. abscessus, including Mycobacterium avium and *Burkholderia* spp. infections. The strategies deployed here, including exploitation of phage libraries of related hosts, phage genome engineering, host range expansion, and growth of induced prophages, can be applied to these and other pathogens to expand the repertoire of therapeutically useful phages. Although the number of useful phages may remain relatively small for some infections, infrequent phage resistance and tradeoffs for *in vivo* fitness should improve their therapeutic utility.

## MATERIALS AND METHODS

### Bacterial strains.

M. smegmatis mc^2^155 is a laboratory stock strain and was grown as previously described (40). M. abscessus ATCC 19977 was obtained from the American Type Culture Collection. M. abscessus clinical isolates were received on Lowenstein-Jensen slants and streaked out on Middlebrook 7H10 agar (Difco) supplemented with oleic acid-albumin-dextrose-catalase (OADC) and 1 mM CaCl_2_ and were grown for 5 to 7 days at 37°C. Liquid cultures were inoculated from a single colony and grown in Middlebrook 7H9 medium with OADC and 1 mM CaCl_2_ for 4 to 5 days at 37°C, with shaking. For plaque assays, M. abscessus cultures were sonicated briefly in a cup-horn sonicator (Q-sonica) as described previously (17). The GD54 strain sequenced and analyzed was GD54H, one of several isolates from the same source. Similarly, the GD35 strain sequenced and analyzed was otherwise designated GD35B. Strains GD43A and B have different numbers of prophages and were therefore analyzed as separate strains. Strain GD43A has six prophages (in clusters MabA, MabB, MabC, MabJ, MabK, and MabL) and GD43B has only four of these (MabA, MabL, MabK, and MabJ). There are no plasmids in either GD43A or GD43B.

### Genome sequencing.

Bacterial and phage genomes were sequenced by Illumina alone or supplemented with Nanopore reads as described previously (41, 42) and details are provided in Table S1. For all strains, Illumina sequencing libraries were prepared from genomic DNA using NEB Ultra II FS kits with dual-indexed barcoding. Libraries were pooled and run on an Illumina MiSeq, yielding 300-base paired-end reads. In some cases, Oxford Nanopore libraries were also constructed from genomic DNA using Rapid Sequencing Barcoding kits, then pooled and run on a MinION device using FLO-MIN106D flowcells. Illumina reads for each strain were trimmed and quality-controlled using Skewer (43). Trimmed Illumina reads were then assembled using Unicycler (44), incorporating Nanopore reads when available.

In the case of complete genomes, assemblies were viewed, stitched, corrected, and finalized using Consed version 29 (45, 46). GraphMap (47) was used to align long Nanopore reads to provisional assemblies and resolve repetitive regions. The first base and orientation of each complete circular chromosome was chosen to match those of the ATCC 19977 strain and/or to align with the first base of the *dnaA* gene. Complete circular plasmids were similarly oriented and cut so that base 1 was the first base of a predicted *repA* gene.

### Phylogenetic trees.

Phylogenies were created using CSI Phylogeny 1.4, a SNP-based concatenated alignment, available on the DTU server (https://cge.cbs.dtu.dk/services/CSIPhylogeny/) (48). Complete and multiple sequence fasta files for 84 genomes were aligned to reference genome (ATCC 19977, CU458896), snp pruning disabled. Newick files were viewed in FigTree v1.4.4 and imported into iTOL (https://itol.embl.de/).

### Prophage identification.

Identification of additional prophages was accomplished by searching with PHASTER (49) for phage-like regions followed by careful manual inspection, identification of *attL* and *attR* attachment sites, and confirmation of the predicted *attB* sequences in the type-strains of either M. abscessus subsp. *abscessus* ATCC 19977, M. abscessus subsp. *massiliense*, or M. abscessus subsp. *bolletii* BD^T^ (23–25). Phamerator (50) databases “Actino_prophage_15” and “Abscessus_prophages_5” were constructed for comparative genomic analyses.

Of the 122 prophages identified, 80 were either in completely sequenced genomes or were wholly within one contig in WGS assemblies (Table S5), and complete genome sequences were extracted. The other 42 were in multiple contigs (Table S5) and, although complete prophage sequences were not available, sufficiently large segments were available to reveal their relationships to other prophages.

Several of the clusters contained only a single prophage member, although database searches suggested that all of these have relatives in other sequenced M. abscessus genomes.

The very extensive diversity of the plasmid genes is reflected in the finding that when sorted into protein phamilies (as described previously), 58% of the genes are “orphams” without closely related genes in this data set.

### Phage infections.

Mycobacterial strains were grown and tested for phage susceptibility as described previously (17). Twenty-three mycobacteriophages were used for the initial screening of M. abscessus clinical isolates. These phages were chosen from clusters known to have expanded host range (40) and others were chosen based on screening completed previously on M. abscessus clinical isolates (17). Phage lysates were 10-fold serially diluted and plated on M. smegmatis mc^2^155 and each M. abscessus clinical isolate. The phiGDxx series of phages were isolated by plating clarified lysates of M. abscessus strains on M. abscessus strains, purifying phages from cleared areas on the sensitive strains, and growing to high titer.

### Phage engineering.

Bacteriophage recombineering of electroporated DNA (BRED) was used to generate the three single base mutations (C12442A, T31726C, and A43657T) of D29, as well as FionnbharthΔ*45*Δ*47* and Faith1Δ*38-40* (51). Oligonucleotides are shown in Table S8 (https://phagesdb.org/documents/categories/15/). For D29, MAMA PCR was used to screen potential mutants. Once pure, they were serially diluted and plated on M. smegmatis mc^2^155 and GD40. For Fionnbharth and Faith1, PCR using flanking primers was used to screen plaques for homogeneous deletion derivatives. All phage mutants were sequenced. See Table S8 (https://phagesdb.org/documents/categories/15/) for a list of oligonucleotides.

### Phage-resistant M. abscessus mutants.

Survival assays of M. abscessus strains sensitive to phages were set up to isolate resistant mutants. An aliquot of 1 ml of ∼1 × 10^8^ CFU/ml of culture was mixed with, or without, one log higher concentration (∼1 × 10^9^ PFU/ml) of phage. These were incubated at 37°C with shaking, and 100 μl was plated after 2 and 5 days. Plates were grown at 37°C for 5 to 7 days and then photographed. Colonies from these plates were picked, streaked twice, and then grown in liquid culture to be used in top agar overlays for phage-resistance screening. Top agar overlays were spotted with serial dilutions of the phage of interest and incubated at 37°C for 5 to 7 days.

### phiGDxx phages.

Log phase liquid cultures of clinical isolates were grown, and one ml of each culture was centrifuged at 14,000 × *g* for 2 min. The supernatant (from each “donor” strain) was saved and used for spotting on top agar overlays of other clinical isolates (the “recipient” strain). These plates were incubated at 37°C for 5 to 7 days. Any clearing on the overlay where a supernatant was spotted was picked, using a sterile pipet tip, into phage buffer (10 mM Tris-HCl [pH 7.5], 10 mM MgSO_4_, and 68 mM NaCl). The phiGDxx phages were purified and then amplified on the receiving strain. DNA was extracted from lysates of phiGDxx phages using a standard phenol-chloroform/EtOH precipitation protocol.

### Data availability.

The completed and WGS genome sequencing data for M. abscessus clinical isolates have been submitted to GenBank and accession numbers are listed in Table S1. The phiGDxx genomes have the following GenBank and accession numbers: phiGD89-1 (MW314851), phiGD57-1 (MW314852), phiGD34-2 (MW314853), phiGD24-3 (MW314854), phiGD23-1 (MW314855), phiGD22-1 (MW314856), phiGD21-1 (MW314857), phiGD20-1 (MW314858), phiGD17-1 (MW314859).
