# Supplementary material for: Mycobacterium abscessus Strain Morphotype Determines Phage Susceptibility, the Repertoire of Therapeutically Useful Phages, and Phage Resistance
Source: mBio. 2021 Mar 30;12(2):e03431-20. doi: 10.1128/mBio.03431-20 (PMC8092298; doi:10.1128/mBio.03431-20)
Supplement: TABLE S2 [file mBio.03431-20-st002.pdf]

Table S2. Genomic differences in smooth and rough *Mycobacterium abscessus* clinical isolates

Strain	Subsp[1]	R or S[2]	Differences[3]	ATCC19977 coordinates[4]	Comments
GD43A	a	S	wt	wt	
GD43B	a	R	1 bp change, stop in *mps1*	G4158543C	
GD68A	m	R	1 bp insertion in *mps1*	1 bp insertion at 4154628	
GD68B	m	S	wt	wt	
GD69A	a	R	wt	wt	
GD69B	a	S	Two changes elsewhere in genome	No changes in this region	
GD100A	a	S	wt	wt	
GD100B	a	R	1 bp change causing Y321C in *mps2*	T4149638C	
GD01	a	R	1 bp insertion in *mmpL4a*	1 bp insertion at 4180153	
GD02	m	R		Some bp differences	
GD05	a	R	3 bp insertion in *mps2*	3 bp insertion at 4147522	
GD08	a	R	2 bp deletion in *mps2*	2 bp deletion at 4144550	
GD10	m	R	1 bp deletion in *mps1*	1 bp deletion at 4154573	
GD11	a	R	1 bp insertion in *mps1*	1 bp insertion at 4153951	
GD12	a	R			Only part seq
GD13	a	R	3430 bp deletion in *mps1*	3430 bp del 4155216 - 4158646	
GD14	a	R		1 bp difference	
GD15	a	R	2 bp insertion in *mps1*	2 bp insertion at 4150877	Same as GD41
GD17	a	R	None identified		
GD19	a	R	35 bp insertion in *mps1*	35 bp insertion at 4157643	
GD20	a	R	2 1bp substitutions; *mps2*, 4105c	G4150037A; A4165005G	
GD22	a	R	1 bp deletion in *mps2*	1 bp deletion at 4149483	
GD23	a	R		1 bp difference	
GD24	a	R	68 bp duplication in *mps1*	68 bp dup of 4159794 - 4159898	
GD25	a	R	12 bp deletion in *mps2*	12 bp deletion 4150491-4150502	
GD26	a	R	1 bp deletion in *mps1*	1 bp deletion at 4156043	
GD27	a	R		1 bp difference	
GD30	m	R		Minor bp differences	
GD35	a	R	1 bp deletion in *mps1*	1 bp deletion at 4160455	Same as GD38
GD38	a	R	1 bp deletion in *mps1*	1 bp deletion at 4160455	Same as GD35
GD40	a	R		Three base differences	
GD41	a	R	2 bp insertion in *mps1*	2 bp insert at 4150877	Same as GD41
GD45	a	R	1 bp insertion in *mps1*	1 bp deletion at 4159934	
GD51	a	R	1 bp insertion in *mps1*	1 bp insertion at 4159093	
GD52	a	R	2 bp deletion in *mps1*	2 bp deletion at 4154035	
GD54	a	R	1 bp del in *mps1*	1bp del 4160654	Same as GD86
GD56	a	R			Only part seq
GD57	a	R	1 bp ins in *mps2*	1 bp insertion at 4144093	
GD59	a	R		Minor bp differences	
GD60	m	R	1 bp insertion in *mps2*<br>1 bp deletion in *mmpL4b*	1 bp insertion at 4146273<br>1 bp deletion at 4177862	
GD62	a	R			Only part seq
GD79	m	R	1 bp insertion in *mps1*	1 bp insertion at 4152255	
GD82	m	R	2 bp deletion in *mps2*	2 bp deletion at 4146637	
GD86	a	R	1 bp del in *mps1*	1 bp del 4160654	Same as GD54
GD87	a	R	Many (>40) bp differences		
GD88	a	R	None identified		
GD89	a	R	1 bp insertion in *mps2*	1 bp insertion at 4145405	
GD92	a	R			Only part seq
GD95	a	R	1 bp deletion in *mps1*	1 bp deletion at 4152166	
GD102	a	R	1 bp insertion in *mps2*	1 bp insertion at 4144781	
GD104	m	R		Several bp differences	
GD111	m	R	1 bp deletion in *mps1*	1 bp deletion at 4151505	

[1]Subspecies designated: a, *abscessus*; m, *massiliense*.
[2]Rough (R) or Smooth (S) colony morphotype
[3]Genes affected by mutations in the region MAB_4117c (*mmpS*) through MAB_4097c (*gap*). Differences were identified by sequence comparison of ~40 kbp spanning this region with that of *M. abscessus* ATCC19977.
[4]Coordinates for mutations are shown corresponding to *M. abscessus* ATCC19977 (Accession #CU45896)
