# Supplementary material for: Mycobacterium abscessus Strain Morphotype Determines Phage Susceptibility, the Repertoire of Therapeutically Useful Phages, and Phage Resistance
Source: mBio. 2021 Mar 30;12(2):e03431-20. doi: 10.1128/mBio.03431-20 (PMC8092298; doi:10.1128/mBio.03431-20)
Supplement: TABLE S1 [file mBio.03431-20-st001.pdf]

Table S1. Sequencing details of *Mycobacterium abscessus* clinical isolates

Strain[1]	Length (bp)[2]	Contigs[3]	Coverage[4]	Status[5]	GenBank Accession[6]
GD01A	4901034	1	421	Complete	CP035923
GD01B	4889373	1	180	Complete	CP035924
GD02	5000184	27	402	WGS	JADLKQ000000000
GD03	5010693	35	459	WGS	JADWWQ000000000
GD04	5120596	43	547	WGS	JADWWR000000000
GD05	5352395	1	88	Complete	CP065287
GD08	5192767	52	81	WGS	JADWWS000000000
GD09	5036145	76	52	WGS	JADWWT000000000
GD10	5073834	92	92	WGS	JADLKR000000000
GD11	5195782	50	64	WGS	JADWWU000000000
GD12	5086633	52	98	WGS	JADWWV000000000
GD13	5269564	55	72	WGS	JADWWW000000000
GD14	5022135	44	88	WGS	JADWWX000000000
GD15	5067256	48	55	WGS	JADWWY000000000
GD16	5008827	56	126	WGS	JADWWZ000000000
GD17	5107403	1	85	Complete	CP063165
GD18	5002397	70	60	WGS	JADWXA000000000
GD19	4915323	1	64	Complete	CP063328, CP063329
GD20	5026147	1	52	Complete	CP063327
GD21	5218527	1	107	Complete	CP065284, CP065285, CP065286
GD22	5073923	1	51	Complete	CP063324, CP063325, CP063326
GD23	5093402	47	63	WGS	JADLKS000000000
GD24	5162219	43	54	WGS	JADLKT000000000
GD25	5112318	1	70	Complete	CP063320, CP063321, CP063322, CP063323
GD26	5098671	1	86	Complete	CP063319
GD27	5067484	44	95	WGS	JADLKU000000000
GD28	4812590	36	54	WGS	JADLKV000000000
GD30	4980908	39	56	WGS	JADWXB000000000
GD33	5144027	62	59	WGS	JADLKW000000000
GD34	5373015	66	68	WGS	JADLKX000000000
GD35	5051409	46	119	WGS	JADLKY000000000
GD36	5430883	110	67	WGS	JADWXC000000000
GD37	4755296	48	67	WGS	JADLKZ000000000
GD38	5182416	1	66	Complete	CP063318
GD39	5111964	52	71	WGS	JADWXD000000000
GD40	5043795	44	115	WGS	JADWXE000000000
GD41	5074858	1	75	Complete	CP065283
GD42	5197760	1	54	Complete	CP065280, CP065281, CP065282
GD43A	5377795	1	93	Complete	CP065279
GD43B	5286951	1	88	Complete	CP065278
GD44	4906511	23	82	WGS	JADWXF000000000
GD45	5079188	46	99	WGS	JADLLA000000000
GD47	5084415	45	98	WGS	JADWXG000000000
GD51	5076192	51	59	WGS	JADWXH000000000
GD52	5342162	64	68	WGS	JADLLB000000000
GD53	5237166	39	73	WGS	CP065033
GD54	5155522	1	125	Complete	CP065276, CP065277
GD55	4990177	44	72	WGS	JADWXI000000000
GD56	5094848	56	86	WGS	JADWXJ000000000
GD57	5120346	1	79	Complete	CP065275
GD58	5136259	43	75	WGS	JADWXK000000000
GD59	5073623	1	71	Complete	CP065274
GD60	5178709	118	77	WGS	JADWXL000000000
GD61	5090086	60	71	WGS	JADWXM000000000
GD62	5369842	65	64	WGS	JADWXN000000000
GD63	4733675	121	106	WGS	JADWXO000000000
GD64	4862420	42	206	WGS	JADWXP000000000
GD68A	5119772	1	92	Complete	CP065273
GD68B	5119711	1	62	Complete	CP065272
GD69A	5162653	1	77	Complete	CP065269, CP065270, CP065271
GD69B	5174676	1	108	Complete	CP065266, CP065267, CP065268
GD72	5132444	162	51	WGS	JADWXQ000000000

GD75	5017220	101	44	WGS	JADWXR000000000
GD79	4924989	52	47	WGS	JADLLC000000000
GD81	5174487	53	86	WGS	JADWXS000000000
GD82	4946210	40	78	WGS	JADWXT000000000
GD84	5097571	53	55	WGS	JADWXU000000000
GD85	4980587	84	65	WGS	JADWXV000000000
GD86	5263250	51	92	WGS	JADWXW000000000
GD87	5055936	136	71	WGS	JADWXX000000000
GD88	4772874	70	50	WGS	JADWXY000000000
GD89	5055865	48	103	WGS	JADWXZ000000000
GD90	4822336	85	51	WGS	JADWYA000000000
GD91	5312857	1	99	Complete	CP065265
GD92	5022095	297	80	WGS	JADWYB000000000
GD95	5140392	106	51	WGS	JADWYC000000000
GD97	4753657	38	59	WGS	JADWYD000000000
GD100A	5136515	1	134	Complete	CP065263, CP065264
GD100B	5136230	1	117	Complete	CP065183, CP065184
GD102	5261854	56	92	WGS	JADWYE000000000
GD104	5099351	33	76	WGS	JADWYF000000000
GD108	5075504	52	67	WGS	JADWYG000000000
GD111	5112275	86	66	WGS	JADWYH000000000

[1]Strains are given a GDXX designation; multiple isolates from the same patient are designated GDXXA, GDXXB, etc.
[2]For complete genomes, the length is the precise length of the bacterial chromosome. For WGS genomes, length is the sum of the lengths of all assembled contigs and therefore may be smaller or larger than the true genome size.
[3]For WGS genomes, the number of contigs obtained through sequencing and assembly with Unicycler.
[4]Genome coverage obtained through WGS sequencing for each strain.
[5]Genome sequencing with Illumina whole genome sequencing or Illumina and Nanopore to completion.
[6]Main chromosome accession numbers are provided first, followed, if applicable, by plasmid accession numbers.
