# Supplementary material for: Mycobacterium abscessus Strain Morphotype Determines Phage Susceptibility, the Repertoire of Therapeutically Useful Phages, and Phage Resistance
Source: mBio. 2021 Mar 30;12(2):e03431-20. doi: 10.1128/mBio.03431-20 (PMC8092298; doi:10.1128/mBio.03431-20)
Supplement: TABLE S3 [file mBio.03431-20-st003.pdf]

Table S3. *M. abscessus* survival following phage challenge

Strain	M[1]	Phage challenge[2]	# of Survivors[3]	Total tested[4]	FR[5]	PR[6]
GD01	R	BPsΔ*33*HTH_HRM10 + Muddy + ZoeJΔ*45*	6	6	6;0;6	0;5;0
GD17	R	BPsΔ*33*HTH_HRM^{GD03}	<10	4	0	1
GD19	R	Muddy	<10	6	2*	1*
GD20	R	BPsΔ*33*HTH_HRM10	<10	4	0	0
GD22	R	BPsΔ*33*HTH_HRM10	<30	4	4	0
		BPsΔ*33*HTH_HRM^{GD03}	<30	2	0	2
		Itos	lawn	-	-	-
		BPsΔ*33*HTH_HRM10 + Itos	<40	3	2;0	1;3
		BPsΔ*33*HTH_HRM^{GD03} + Itos	<40	4	1;1	3;0
GD24	R	BPsΔ*33*HTH_HRM10	<40	-	-	-
		BPsΔ*33*HTH_HRM^{GD03}	<10	3	0	0
GD25	R	Muddy	<10	3	1	0
GD26	R	BPsΔ*33*HTH_HRM10	>160	8	1	1
		BPsΔ*33*HTH_HRM^{GD03}	>200	4	0	0
GD27	R	BPsΔ*33*HTH_HRM10	0	-	-	-
		BPsΔ*33*HTH_HRM^{GD03}	0	-	-	-
		Itos	lawn	-	-	-
		BPsΔ*33*HTH_HRM10 + Itos	0	-	-	-
		BPsΔ*33*HTH_HRM^{GD03} + Itos	0	-	-	-
GD30	R	Muddy	lawn	-	-	-
GD35	R	BPsΔ*33*HTH_HRM10	<10	4	1	0
		BPsΔ*33*HTH_HRM^{GD03}	<10	4-DNG	-	0
GD38	R	BPsΔ*33*HTH_HRM10	<10	3	3	0
		BPsΔ*33*HTH_HRM^{GD03}	<10	1	1	0
		FionnbharthΔ*45*Δ*47*	lawn	-	-	-
GD40	R	BPsΔ*33*HTH_HRM10	<20	4-DNG	-	-
		BPsΔ*33*HTH_HRM^{GD03}	<20	4	0	4
		D29	lawn	-	-	-
GD41	R	BPsΔ*33*HTH_HRM10	<10	4	3	0
		BPsΔ*33*HTH_HRM^{GD03}	<10	4	2	0
		Itos	lawn	-	-	-
		BPsΔ*33*HTH_HRM10 + Itos	<10	4-DNG	-	-
		BPsΔ*33*HTH_HRM^{GD03} + Itos	<10	4	4;2	0;0
GD43B	R	BPsΔ*33*HTH_HRM^{GD03}	0	-	-	-
GD45	R	Muddy	0	-	-	-
GD51	R	Muddy	0	-	-	-
GD52	R	BPsΔ*33*HTH_HRM^{GD03}	<20	4-DNG	-	-
		Itos	lawn	-	-	-
		FionnbharthΔ*45*Δ*47*	<20	4-DNG	-	-
GD54	R	BPsΔ*33*HTH_HRM^{GD03}	?	-	-	-
		Muddy	<10	4	0	0
		BPsΔ*33*HTH_HRM^{GD03} + Muddy	<10	0	-	-
GD57	R	BPsΔ*33*HTH_HRM^{GD03}	<10	4	2	2
		Itos	lawn	-	-	-
		BPsΔ*33*HTH_HRM^{GD03} + Itos	<10	2	1;0	0;1
GD59	R	BPsΔ*33*HTH_HRM^{GD03}	<20	3	3	0
		BPsΔ*33*HTH_HRM10	>20	4	4	0
		Itos	lawn	4	4	0
		BPsΔ*33*HTH_HRM^{GD03} + Itos	<20	3	3;0	0;0
		BPsΔ*33*HTH_HRM10 + Itos	<20	4	4;0	0;0
GD68A	R	Muddy	<10	4	0	0
GD79	R	BPsΔ*33*HTH_HRM^{GD03}	0	-	-	-
		Muddy	0	-	-	-
		ZoeJΔ*45*	0	-	-	-
GD82	R	Muddy	<10	4	1	0

		BPsΔ*33*HTH_HRM10	<10	NT	-	-
		ZoeJΔ*45*	<1000	4	1	0
		BPsΔ*33*HTH_HRM10 + Muddy + ZoeJΔ*45*	<50	4	0;0;4	0;0;0
GD89	R	D29_HRM^{GD40}	<10	4	1	0
		BPsΔ*33*HTH_HRM10 + D29_HRM^{GD40}	<10	2	0;0	0;0
GD100B	R	Itos	lawn	-	-	-
		Faith1Δ*38-40*	lawn	-	-	-
		BPsΔ*33*HTH_HRM10	<30	IP	-	-
GD102	R	Muddy	<10	4	0	1
GD111	R	Muddy_HRM^{GD04}	<5	IP	-	-
GD16	S	BPsΔ*33*HTH_HRM^{GD03}	lawn	-	-	-
GD21	S	BPsΔ*33*HTH_HRM10	lawn	-	-	-
		BPsΔ*33*HTH_HRM^{GD03}	lawn	-	-	-
GD33	S	BPsΔ*33*HTH_HRM10	lawn	-	-	-
		BPsΔ*33*HTH_HRM^{GD03}	lawn	-	-	-
		Itos	lawn	-	-	-
GD34	S	Faith1Δ*38-40*	lawn	-	-	-
GD58	S	Muddy	lawn	-	-	-
GD64	S	Faith1Δ*38-40*	lawn	-	-	-
GD75	S	BPsΔ*33*HTH_HRM10	lawn	-	-	-
		Faith1Δ*38-40*	lawn	-	-	-
		D29_HRM^{GD40}	lawn	-	-	-
GD81	S	Itos	lawn	-	-	-
		Faith1Δ*38-40*	lawn	-	-	-
GD84	S	Itos	lawn	-	-	-
		Muddy	lawn	-	-	-
GD108	S	Itos	lawn	-	-	-
		Itos + Faith1Δ*38-40*	lawn	-	-	-

[1]Morphology of clinical isolate (R-rough; S-smooth).
[2]Phage(s) incubated with *M. abscessus* cells in assay.
[3]Number of survivors in assay.
[4]Total number of survivors screened for phage susceptibility. DNG; surviving colony did not grow in order to test resistance. NT: Not Tested.
[5]The number(s) of surviving colonies with full resistance (FR) to the phage it was challenged with are indicated. If more than one phage was used for selection, the numbers of mutants fully resistant to each of the individual phages are shown, separated by semicolons and ordered as shown to their left. Asterisk indicates a change of morphology from R to S.
[6]The number(s) of surviving colonies partially resistance (PR) to the phage it was challenged with are indicated. If more than one phage was used for selection, the numbers of mutants partially resistant to each of the individual phages are shown, separated by semicolons and ordered as shown to their left.
