# Supplementary material for: Mycobacterium abscessus Strain Morphotype Determines Phage Susceptibility, the Repertoire of Therapeutically Useful Phages, and Phage Resistance
Source: mBio. 2021 Mar 30;12(2):e03431-20. doi: 10.1128/mBio.03431-20 (PMC8092298; doi:10.1128/mBio.03431-20)
Supplement: TABLE S4 [file mBio.03431-20-st004.pdf]

Table S4. Genomic differences in phage resistant mutants (RMs) of *Mycobacterium abscessus* clinical isolates.

Mutant Strain[1]	Phage Challenge	Morphotype	Resistance[2]	Differences[3]
GD17_RM1	BPsΔ*33*HTH_HRM^{GD03}	R	BPsΔ*33*HTH_HRM$^{GD03\ PR}$(EOP 10^{-4})	G915889T causing G210V in “type I polyketide synthase” (MAB_0939)<br>Two nearby copies of ~570 bp repeat near 1075000 collapsed, causing a 1131 bp deletion, and fusing two porin genes (MAB_1080 and MAB_1081)
GD19_RM3	Muddy	R	Muddy	4bp deletion beginning at 2829501 causing a nonsense mutation in “DNA-directed RNA polymerase omega subunit” (MAB_2822c)
GD19_RM5	Muddy	S	Muddy	Deletion of one copy of a 35bp duplication beginning at 4122508 causing an N-terminal lengthening of 1168 amino acids in “probable non-ribosomal peptide synthetase” (MAB_4099c)<br>Muddy’s genome was present in this sequencing sample, but with the change G21064T (G487W in “minor tail protein” PBI_MUDDY_24) from WT Muddy
GD22_RM1	BPsΔ*33*HTH_HRM^{GD03}	R	BPsΔ*33*HTH_HRM10^{R}<br>BPsΔ*33*HTH_$^{GD03\ R}$<br>Itos^{S}	Insertion of a single A at position 3559519 in “ATP-dependent DNA helicase UvrD2” (MAB_3511c) which changes the final 24 AAs and lengthens the protein by 4 AAs
GD22_RM2	BPsΔ*33*HTH_HRM^{GD03} +Itos	R	BPsΔ*33*HTH_HRM10^{R}<br>Itos^{S}<br>BPsΔ*33*HTH_$^{GD03\ PR}$ (EOP 10^{-4})	T3559580C causing D645G in “ATP-dependent DNA helicase UvrD2” (MAB_3511c)<br>Insertion of one additional copy of a 9bp tandem repeat beginning at 4371238 adding three more threonines to a long threonine chain in “Hsp70 family protein” (MAB_4291)
GD22_RM3	BPsΔ*33*HTH_HRM^{GD03} +Itos	R	BPsΔ*33*HTH_HRM10^{R}<br>Itos^{R}<br>BPsΔ*33*HTH_HRM$^{GD03\ R}$	15bp duplication starting at 416903 in intergenic region between “HAD-IB family hydrolase” and “CpaE-like family protein” (MAB_0431c and MAB_0432)<br>The plasmid pGD22-1 is completely absent
GD22_RM4	BPsΔ*33*HTH_HRM10+ Itos	R	BPsΔ*33*HTH_HRM10^{R}<br>Itos^{PR} (EOP 10^{-3})<br>BPsΔ*33*HTH_HRM^{GD03R}	2bp deletion beginning at 925688 causing a nonsense mutation in “type I polyketide synthase” (MAB_0939)
GD25_RM2	Muddy	R	Muddy^{R}	6bp duplication beginning at 1650493 causing a duplication of DS in “DUF3375 domain-containing protein” (MAB_1490)<br>A1996181T is a silent mutation in “putative oxygenase” (MAB_1866c)<br>A2726634G causing I330V in “ornithine decarboxylase” (MAB_2524)<br>One extra copy of a 33bp tandem repeat (4 copies instead of 3 in WT) beginning at 3439883 causing 11-amino-acid duplication in “ribonuclease III” (MAB_3256c)<br>2bp deletion beginning at 4811880 causing an N-terminal lengthening of 95 amino acids in “MCE family protein” (MAB_4598c)
GD26_RM4	BPsΔ*33*HTH_HRM10	R	BPsΔ*33*HTH_HRM10^{R}<br>BPsΔ*33*HTH_HRM^{GD03R}<br>Itos^{S}	28,459bp deletion from 416194 to 444652 (MAB_0431c to MAB_0460)

[1]Resistant mutant strains are named beginning with the wild type strain from which they were derived, followed by "RMX" for Resistant Mutant and the Xth one that was isolated, and finishing with the phage(s) they are resistant to.
[2]Resistace profile of RM, where [R] is resistant, [S] is sensitive and [PR] is partially resistant and efficiency of plating compared to *M. smegmatis* mc^{2}155 is noted.
[3]Differences from wild type strains are listed using coordinates from the wild type sequence. Wild type nucleotides and amino acids are listed first when describing substitutions.
