# Supplementary figures and images for: Mycobacterium abscessus Strain Morphotype Determines Phage Susceptibility, the Repertoire of Therapeutically Useful Phages, and Phage Resistance
Source: mBio. 2021 Mar 30;12(2):e03431-20. doi: 10.1128/mBio.03431-20 (PMC8092298; doi:10.1128/mBio.03431-20)

# phiGD20-1 (MabA1)

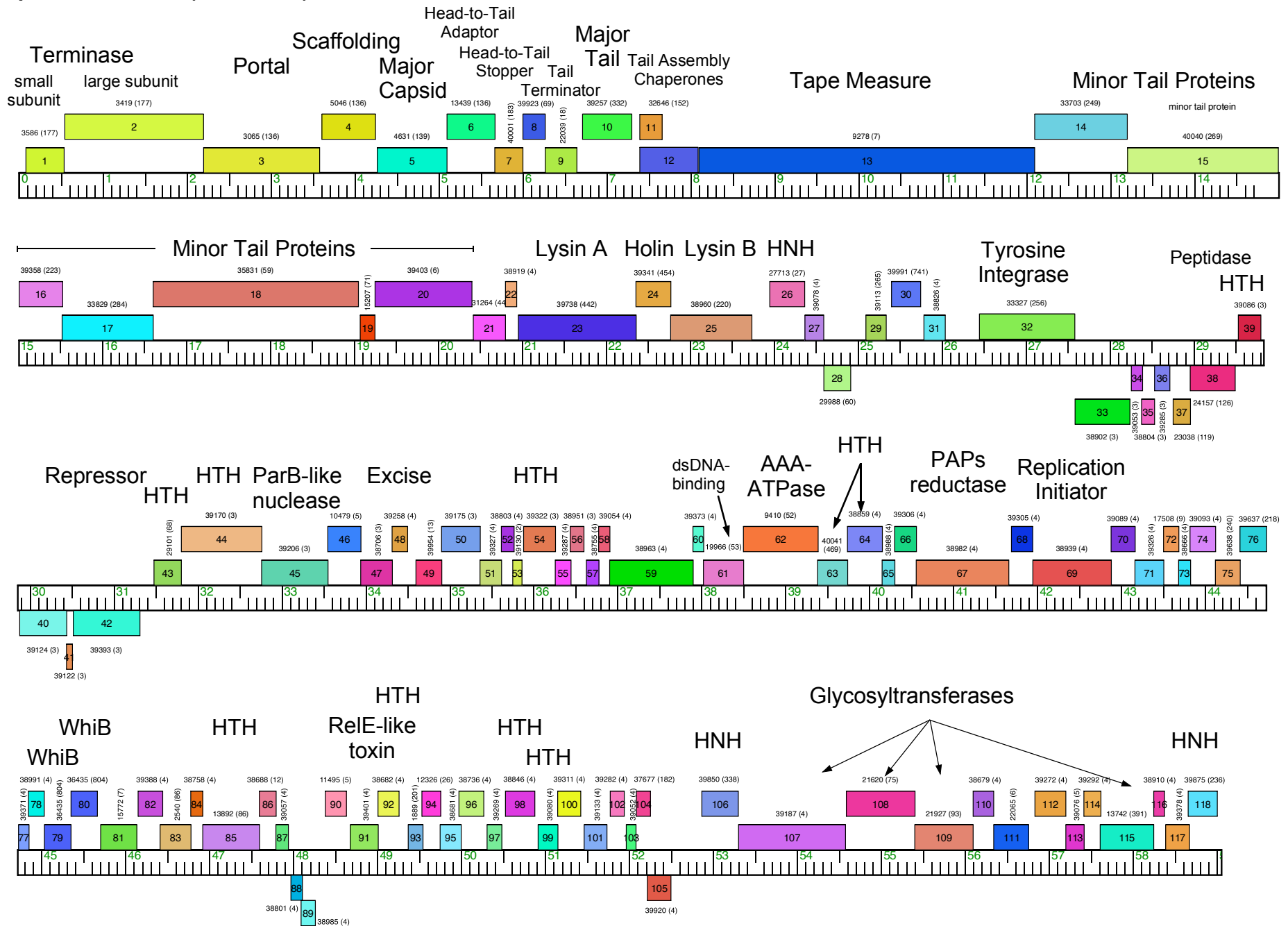

Figure S1

Supplement: FIG S1 [file mBio.03431-20-sf001.pdf]

phiGD21-1 (MabB)

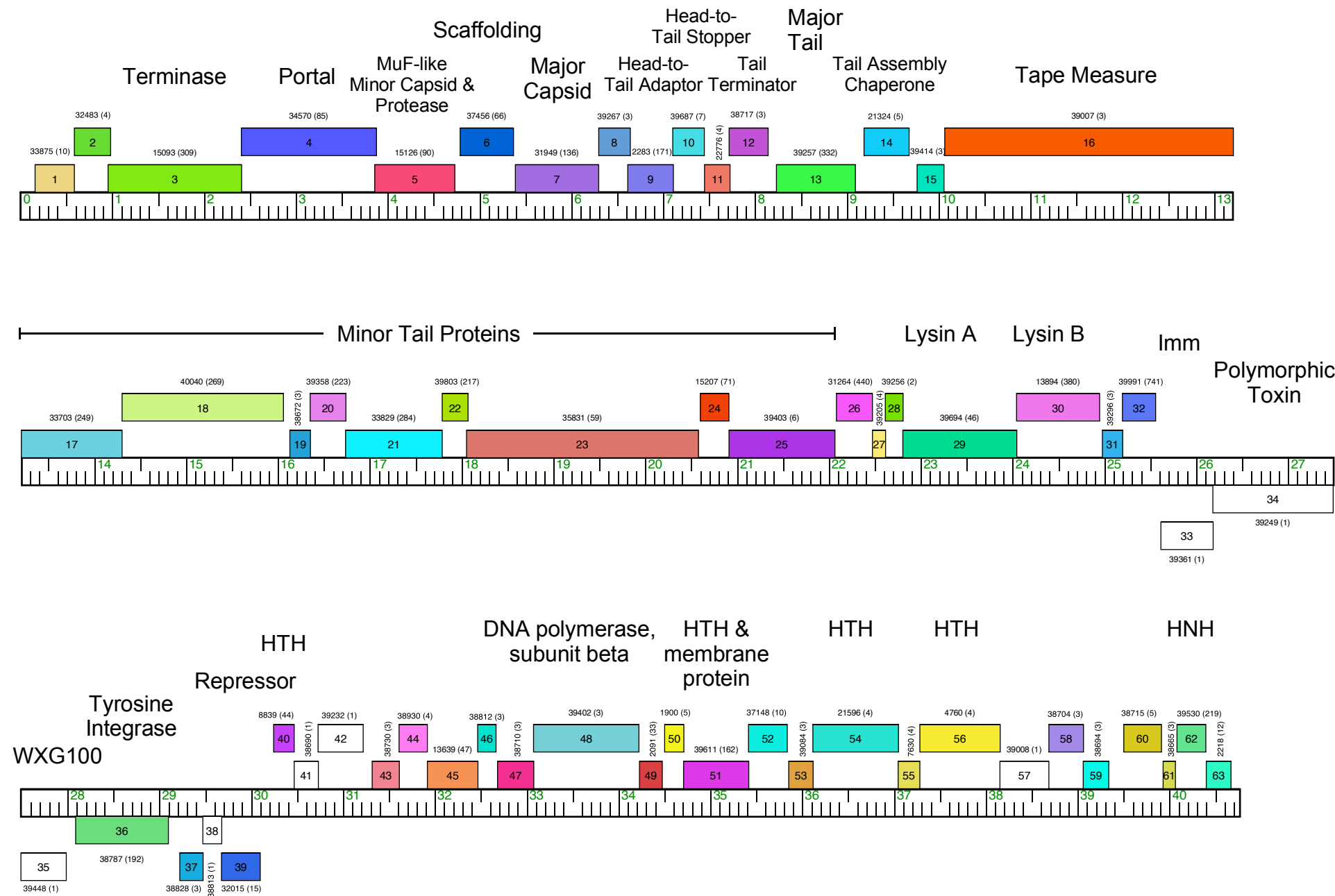

Figure S2

Supplement: FIG S2 [file mBio.03431-20-sf002.pdf]

phiGD17-1 (MabD)

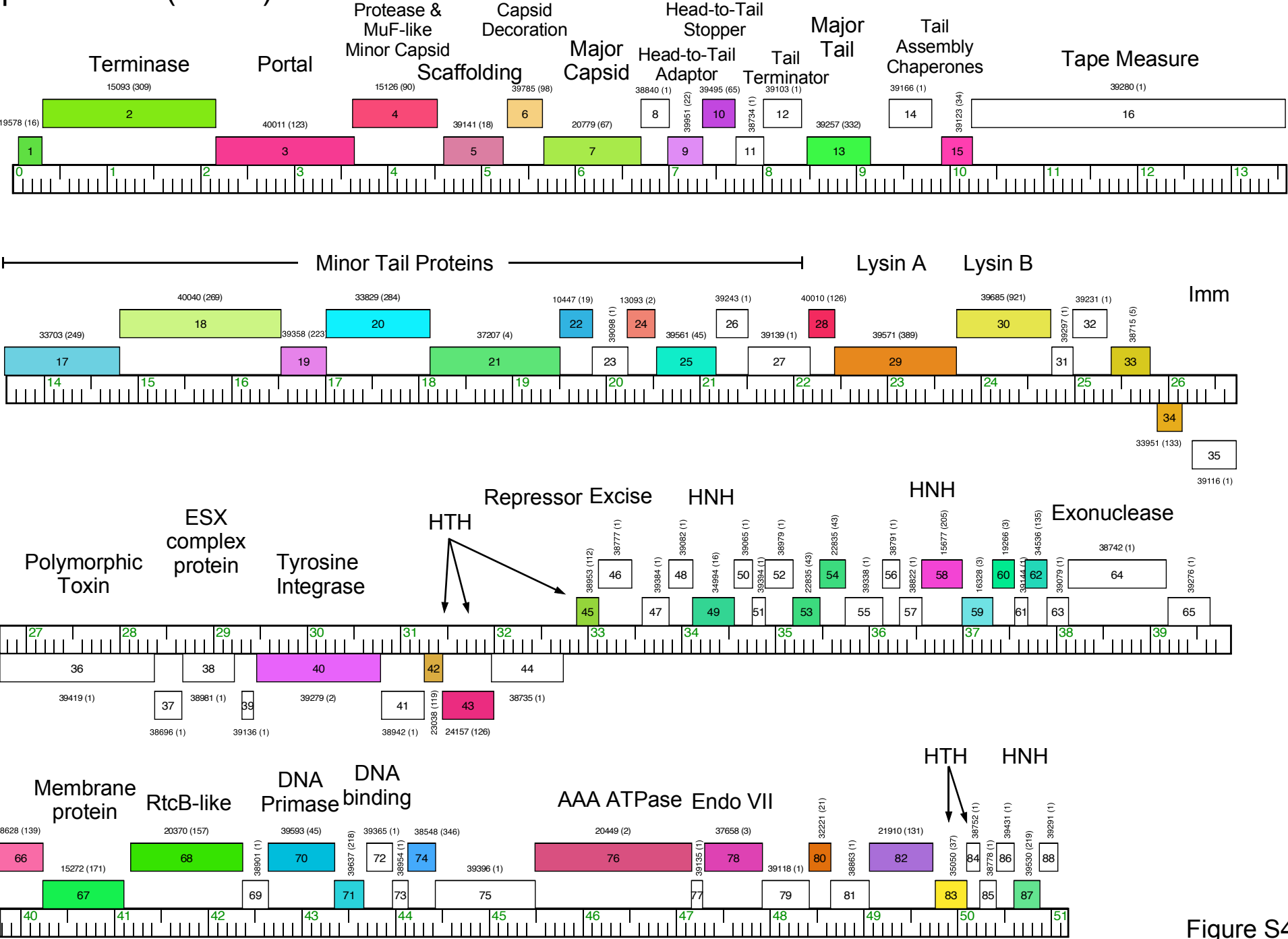

Figure S4

Supplement: FIG S4 [file mBio.03431-20-sf004.pdf]

phiGD24-3 (MabJ)

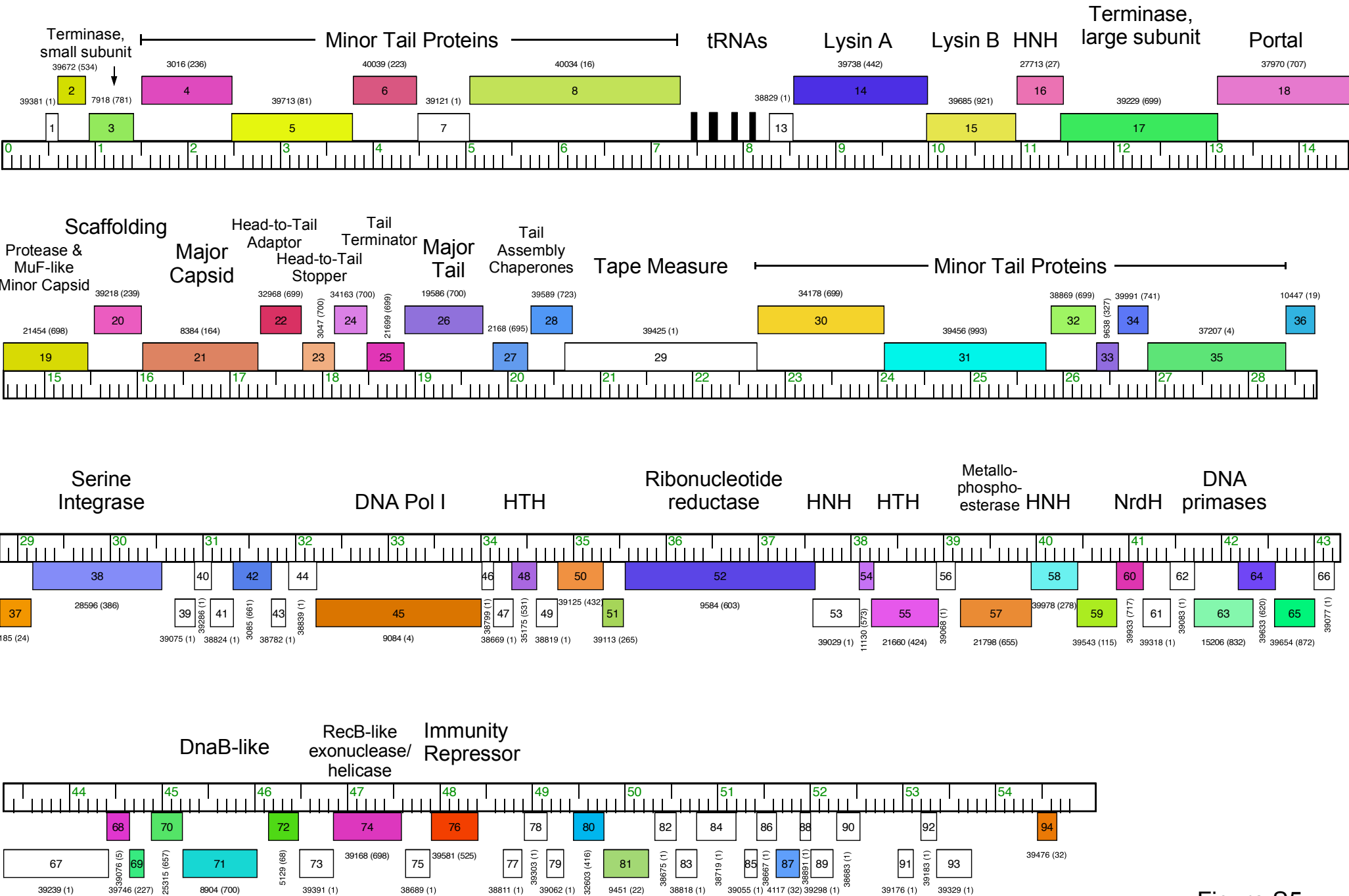

Figure S5

Supplement: FIG S5 [file mBio.03431-20-sf005.pdf]
