# Supplementary material for: Mycobacterium abscessus Strain Morphotype Determines Phage Susceptibility, the Repertoire of Therapeutically Useful Phages, and Phage Resistance
Source: mBio. 2021 Mar 30;12(2):e03431-20. doi: 10.1128/mBio.03431-20 (PMC8092298; doi:10.1128/mBio.03431-20)
Supplement: FIG S3 [file mBio.03431-20-sf003.pdf]

# phiGD57-1 (MabC)

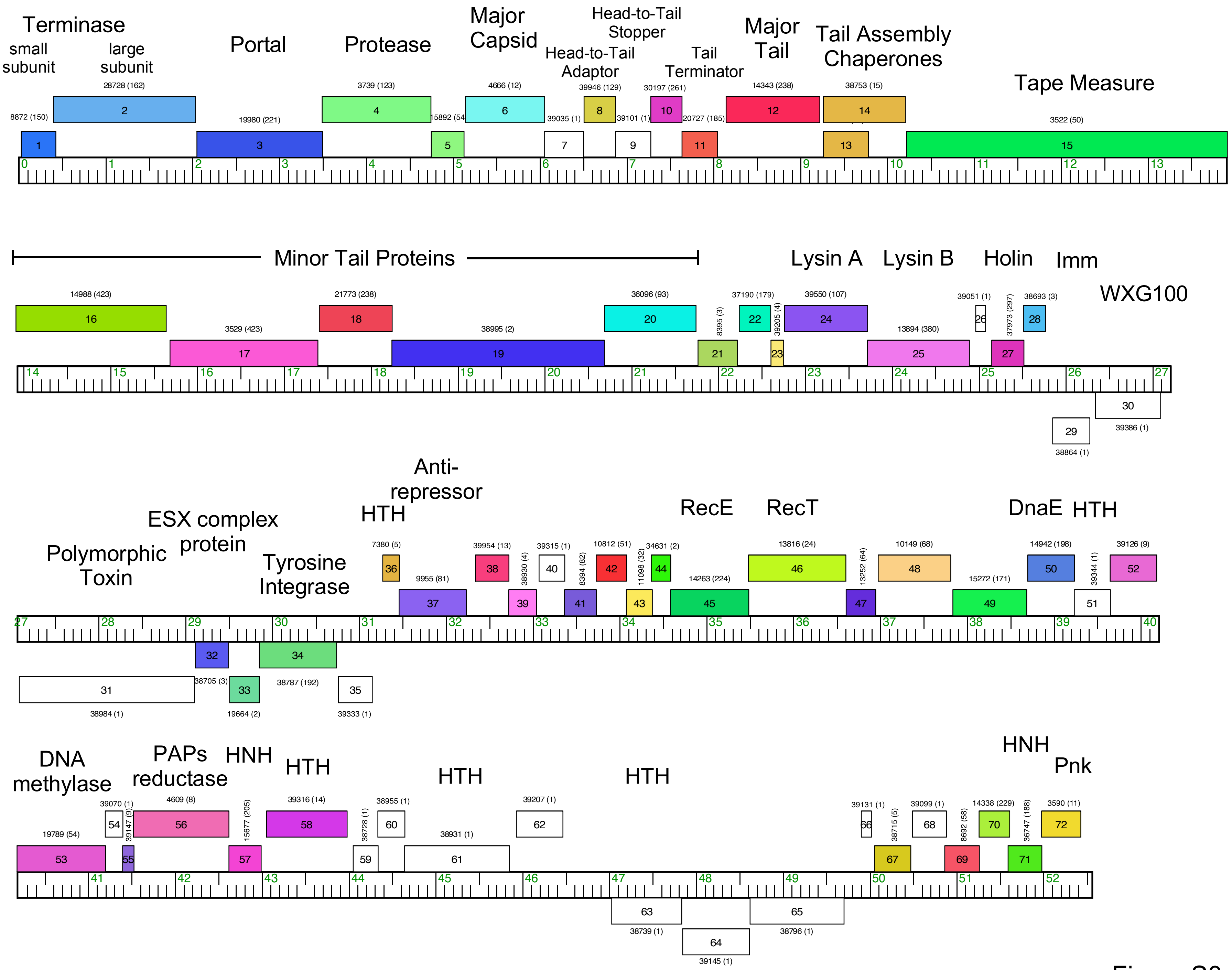


Figure S3
